# Reconstructing interfacial manganese deposition for durable aqueous zinc–manganese batteries

**DOI:** 10.1093/nsr/nwad220

**Published:** 2023-08-16

**Authors:** Yida Hu, Zhexuan Liu, Lanyan Li, Shan Guo, Xuefang Xie, Zhigao Luo, Guozhao Fang, Shuquan Liang

**Affiliations:** School of Materials Science and Engineering, Key Laboratory of Electronic Packaging and Advanced Functional Materials of Hunan Province, Central South University, Changsha 410083, China; School of Materials Science and Engineering, Key Laboratory of Electronic Packaging and Advanced Functional Materials of Hunan Province, Central South University, Changsha 410083, China; School of Science, Hunan University of Technology and Business, Changsha 410205, China; School of Materials Science and Engineering, Key Laboratory of Electronic Packaging and Advanced Functional Materials of Hunan Province, Central South University, Changsha 410083, China; College of Physical Science and Technology, Xinjiang University, Urumqi 830046, China; College of Chemistry, Xiangtan University, Xiangtan 411105, China; School of Materials Science and Engineering, Key Laboratory of Electronic Packaging and Advanced Functional Materials of Hunan Province, Central South University, Changsha 410083, China; School of Materials Science and Engineering, Key Laboratory of Electronic Packaging and Advanced Functional Materials of Hunan Province, Central South University, Changsha 410083, China

**Keywords:** quasi-eutectic electrolyte, aqueous zinc−manganese batteries, solid−liquid interfacial state, mass transfer, charge transfer

## Abstract

Low-cost, high-safety, and broad-prospect aqueous zinc−manganese batteries (ZMBs) are limited by complex interfacial reactions. The solid−liquid interfacial state of the cathode dominates the Mn dissolution/deposition process of aqueous ZMBs, especially the important influence on the mass and charge transfer behavior of Zn^2+^ and Mn^2+^. We proposed a quasi-eutectic electrolyte (QEE) that would stabilize the reversible behavior of interfacial deposition and favorable interfacial reaction kinetic of manganese-based cathodes in a long cycle process by optimizing mass and charge transfer. We emphasize that the initial interfacial reaction energy barrier is not the main factor affecting cycling performance, and the good reaction kinetics induced by interfacial deposition during the cycling process is more conducive to the stable cycling of the battery, which has been confirmed by theoretical analysis, quartz crystal microbalance with dissipation monitoring, depth etching X-ray photon-electron spectroscopy, etc. As a result, the QEE electrolyte maintained a stable specific capacity of 250 mAh g^−1^ at 0.5 A g^−1^ after 350 cycles in zinc−manganese batteries. The energy density retention rate of the ZMB with QEE increased by 174% compared to that of conventional aqueous electrolyte. Furthermore, the multi-stacked soft-pack battery with a cathodic mass load of 54.4 mg maintained a stable specific capacity of 200 mAh g^−1^ for 100 cycles, demonstrating its commercial potential. This work proves the feasibility of adapting lean-water QEE to the stable aqueous ZMBs.

## Teaser

This paper demonstrates a novel quasi-eutectic electrolyte that enables the cycle life of aqueous zinc–manganese batteries by regulating ion behavior and interfacial deposition.

## INTRODUCTION

The solid–liquid interface of an electrode plays a crucial role in the rechargeable battery since it is a key site for electrochemical processes [1–5]. The electrochemical process near to the interface involves the two important steps of mass transfer and charge transfer. Specifically, the ions undergo a mass transfer due to desolvation in the Stern layer, followed by a charge transfer at the electrode surface. However, the adverse competitive reactions of ions at the interface, as well as the deterioration of the interface caused by solid–liquid transition, make it difficult to achieve stability and reversibility on a long-time scale. Therefore, refining the regulation of electrochemical processes at the interface into the regulation of mass transfer and charge transfer is an effective and feasible idea.

Aqueous zinc–manganese batteries (ZMBs) are increasingly being favored as a safe and environmentally-friendly battery candidate [6–14]. However, they have been plagued by capacity degradation when using conventional aqueous electrolytes (e.g. 2 M zinc salt), which are mainly affected by uncontrollable manganese dissolution/deposition interfacial reactions. On the one hand, the indistinguishable solvation structures of hydrated Zn^2+^ and Mn^2+^, without obvious kinetic properties, result in the competitive reaction and co-deposition of a formed product, such as ZnMn_2_O_4_, with low electrochemical activity [15–17]. On the other hand, agglomeration caused by low interface potential will cause uneven and irreversible deposition at the MnO_2_ interface [18], which causes interfacial kinetics deterioration during the cycle process. However, these focal points have received little attention and there is no reliable way to adjust these issues.

Aqueous/organic hybrid electrolytes have been proven easy to adjust the solvation structure of ions [19–23], which, in turn, is expected to adjust the solvation structure of Zn^2+^ and Mn^2+^. However, there are few studies on the matching of these electrolytes with manganese-based materials. Specifically, when the water content is relatively low, it not only inhibits the electrochemical activity of the manganese-based cathode, but is also unfavorable to the interfacial desolvation kinetics of working ions. Developing a new type of aqueous/organic hybrid electrolyte, which is supposed to create the diversity of desolvation kinetics of Zn^2+^ and Mn^2+^ and favorable stable reversible interfacial deposition during cycling, faces an enormous challenge.

**Figure 1.**
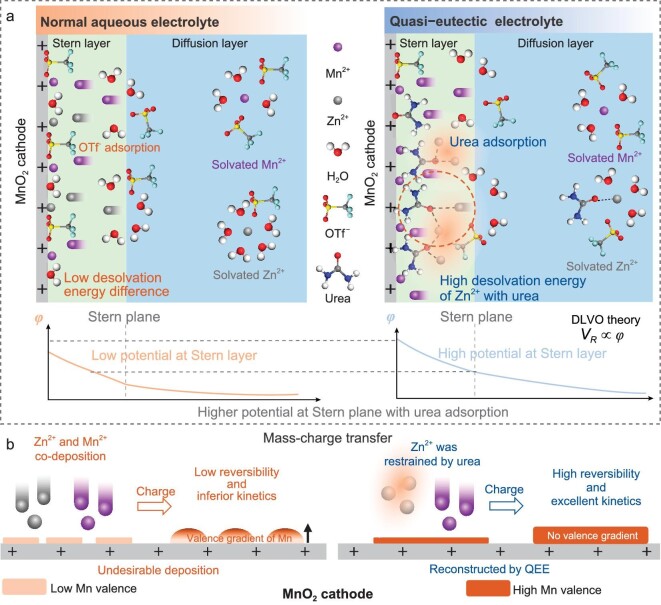
Diagram of the electrochemical behavior of urea-based eutectic electrolyte at the cathode interface. (a) Schematic diagram of the structure and potential, and (b) schematic diagram of mass-charge transfer. *φ* represents the potential at different distances from the cathode interface.

In this work, we construct a lean-water quasi-eutectic electrolyte (QEE) that can promote stable operation of ZMBs. Figure 1a shows that the QEE regulates both the double layer structure of the MnO_2_ cathode and ion solvation structure. In QEE, quartz crystal microbalance with dissipation monitoring (QCM-D) confirmed the substitution of OTf^−^ by urea, which converts the cathode interface from anion enrichment to molecular enrichment. High binding force between Zn^2+^ and urea makes the desolvation of Zn^2+^ at the cathode interface more difficult, thus restraining the mass transfer of Zn^2+^. As shown in Fig. 1b, the modulation reduces the Zn^2+^ deposition at the interface, thus improving the reversibility of the deposition, which was demonstrated with the depth etching XPS profile. At the same time, enrichment of the molecular interface increases the Stern layer potential thus increasing the repulsive force *V_R_* at the interface, alleviating deposition agglomeration. Moreover, optimization of charge transfer and mass transfer at the cathode interface by modulating the interface morphology and the depositions result in highly reversible cathode deposition with excellent kinetics in a long cycle process. As a result, aqueous ZMBs with QEE have demonstrated a stable cycle for over 350 cycles at 0.5 A g^−1^, maintaining a specific capacity of 250 mAh g^−1^. Specifically, a multi-stacked soft-pack battery with a cathodic mass load of 54.4 mg exhibits superior performance, cycling for 100 cycles at 0.3 A g^−1^ while maintaining a specific capacity of 200 mAh g^−1^, demonstrating the practical potential of QEE.

## RESULTS AND DISCUSSION

### Analysis of the structural feature of QEE

**Figure 2.**
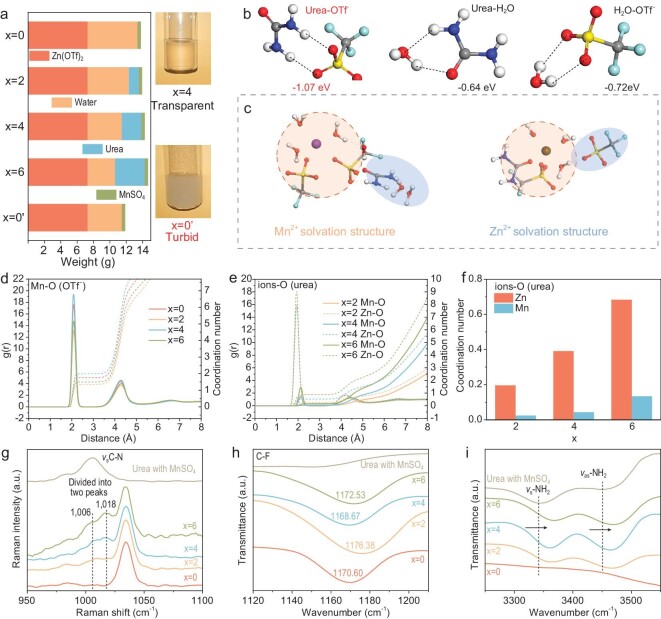
Feature of QEE and its ionic solvation structure. (a) Composition of electrolytes and dissolution condition. (b) The binding energy between different groups. (c) Ionic solvation structure by MD calculations of Mn^2+^ (left) and Zn^2+^ (right). RDF for (d) Mn−O (OTf^−^) pairs and (e) ion−O (urea) pairs. (f) The coordination number of ions−O (urea) pairs. (g) Raman spectra of electrolytes at 950−1100 cm^−1^ representing the stretching vibration of C−N and FTIR spectra of electrolytes at (h) 1120−1210 cm^−1^representing the stretching vibration of C−F and (i) 3250−3550 cm^−1^ representing the stretching vibration of−NH_2_.

In this work, the components of QEE are 2 M Zn(OTf)_2_, high content of urea (4 M and higher) and 0.25 M MnSO_4_. The 2 M Zn(OTf)_2_ + *x* M urea + 0.25 M MnSO_4_ (named as *x* = 0, 2, 4, 6 electrolytes, respectively) and the quality of each component of different electrolytes (total volume 10 ml) is shown in Fig. 2a. The QEE has less water content (∼25 wt.%) than the traditional 2 M ZnSO_4_ electrolyte (∼75 wt.%), such as the water ratio of 29.40 wt.% in the *x* = 4 electrolyte. The addition of urea is conducive to the dissolution of Zn(OTf)_2_ salt due to the hydrogen bond between urea and OTf^−^. Contrary to the transparent *x* = 4 electrolyte, the electrolyte with no urea and the same water content as *x* = 4 electrolyte (named *x* = 0’) presents a turbid state, which proved the eutectic feature of *x* = 4 electrolyte.

It should be noted that the eutectic electrolytes play an important role in constructing different ionic solvation sheaths for Zn^2+^ and Mn^2+^, which has not been discussed previously. According to the DFT results (Fig. 2b, Table S1), the lower binding energy between OTf^−^ and urea than other solvent species indicates the formation of more stable hydrogen bonds. NMR also demonstrated the interaction of OTf^−^ with urea in the electrolytes (Fig. S1). The molecular dynamic (MD) calculations (Fig. S2) show that the urea molecules do not enter the inner solvation structure of Mn^2+^, but does enter the inner solvation structure of Zn^2+^ (Fig. 2c). The results show that urea molecules can affect the desolvation behavior of these two ions, which is also the reason for their different electrochemical behaviors. It can be observed from radial distribution function (RDF) in Fig. 2d and Fig. S3 that both Zn^2+^ and Mn^2+^ tend to combine more tightly to OTf^−^ and more loosely to H_2_O (Fig. S4) as the urea concentration rises. It is noteworthy that the binding of Mn^2+^ to OTf^−^ is strongest when the urea concentration is 4 M (*x* = 4), reducing the charge density of the overall solvation structure. However, the binding of Mn^2+^ to urea is very weak, irrespective of the urea concentration (Fig. 2e). In contrast, the binding of Zn^2+^ to urea becomes progressively stronger as the urea concentration rises, which is also demonstrated in Fig. 2f. In addition, a number of theoretical calculations were used to further investigate the solvation structure. In Fig. S5, the LUMO and HOMO of solvation structures of Zn^2+^ and Mn^2+^ in *x* = 0 and *x* = 4 electrolytes were both calculated. For different electrolytes, the gap between LUMO and HOMO did not change for the three solvation structures, proving that the entry of urea into the solvation structure does not affect the stability of the solvation structure itself, and that the stability of the solvation structure is consistent for Zn^2+^ and Mn^2+^. In Fig. S6, the electrostatic potential of different solvation structures was tested. It can be seen that the electrostatic potential of the solvation structure of Zn^2+^ does not change significantly after the addition of urea. This indicates that the difference in kinetics when the desolvation of the solvation structure is caused by the interface rather than itself.

Raman and FTIR spectra were further conducted to demonstrate the solvation structure of Zn^2+^ and Mn^2+^ in QEE. The peak at ∼1006 cm^−1^ in Raman spectra represents the stretching vibrations of C−N [24], which was divided into two peaks in QEE compared to the pure urea solution (Fig. 2g). This represents a compression of partial C−N and indicated the presence of hydrogen bonds between the −NH_2_ group and the hydrogen bond acceptor OTf^−^. As the concentration of urea increases, it is reasonable that the increased peak strength indicates that more abundant hydrogen bonds are available. In the FTIR spectra, the peak at ∼1035 cm^−1^ and ∼1170 cm^−1^ represents the vibration of S = O and C−F [25,26]. As the concentration of urea increased, the S = O peak appears redshift, implying that OTf^−^ was gradually moving into the solvation structure of the cation (Fig. S7). The C−F peak shows the most obvious redshift at the urea concentration of 4 M, which means the strongest hydrogen bond interaction between the C−F group and the −NH_2_ group (Fig. 2h). Similarly, the two strong bands in the high FTIR spectra range, which could be assigned to the −NH_2_ stretching vibrations, shift to a higher wavenumber compared to the urea solution as shown in Fig. 2i. This also indicated that QEE has a stronger hydrogen bond than those single-component electrolytes [27]. In addition, in the high wavenumber region of Raman spectra, the intensity of peaks representing the stretching vibration of −NH_2_ at ∼3247 cm^−1^, ∼3393 cm^−1^ and 3492 cm^−1^ [27] increases in line with the increase in urea concentration (Fig. S8). This proves that the urea is completely dissolved in the electrolyte and the greater the −NH_2_ group in the electrolyte, the more provision for stronger hydrogen bonding. As a result, combining the coordination preferences of Zn^2+^ and Mn^2+^, it can be considered that both urea and OTf^−^ could construct a direct coordination interaction with Zn^2+^, while in the OTf^−^−dominated Mn^2+^ solvation structure, the urea can only be located in the outer sheath.

In summary, the QEE was prepared based on the abundant hydrogen bonds between urea and OTf^−^, which decreased the water content in the electrolyte and cation solvation sheath. More importantly, the semi-full 3d orbitals of Mn^2+^ allow the species with higher polarizability to participate in its solvation structure, while the Zn^2+^ shows an increased tendency to coordinate with urea [28,29]. Such a different ionic solvation structure lays the basis for the difference in mass transfer between Zn^2+^ and Mn^2+^ at the cathode interface, which will be elaborated on later.

### Cathodic EDL structure in QEE

**Figure 3.**
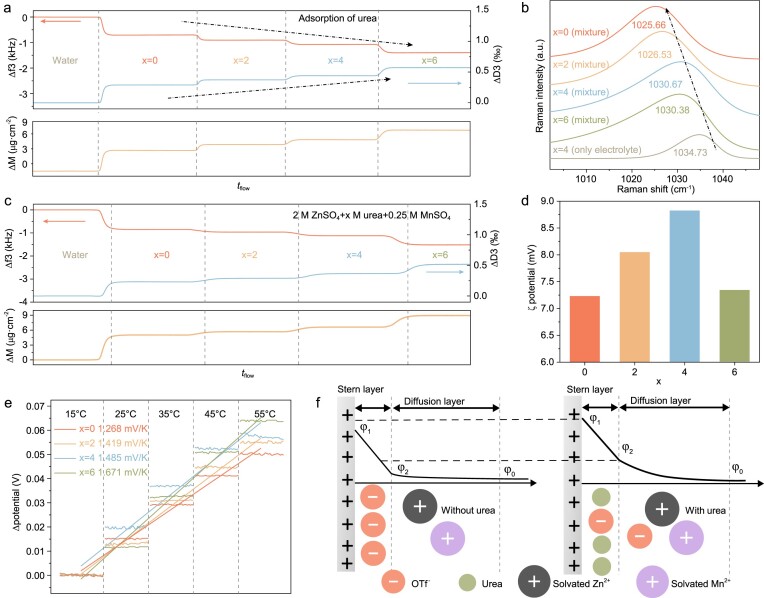
Cathodic interfacial ion adsorption and induced EDL structure. (a) QCM-D curves of OTf^−^-based electrolytes. (b) Raman spectra of the electrolyte-MnO_2_ mixture. (c) QCM-D curves of SO_4_^2−^-based electrolytes. (d) ζ potential of MnO_2_ in electrolytes after 10-fold dilution. (e) Potential-temperature curves. (f) Schematic diagram of the cathode interface.

The electrochemical process of Mn dissolution/deposition reaction is not only affected by the initial energy determined by the ionic solvation structure, the reaction energy barrier caused by the formation and destruction of bonds in the EDL structure at the cathode interface also needs to be highlighted. Quartz crystal microbalance with dissipation monitoring (QCM−D) has been proven to be an effective tool to investigate *in-situ* interactions between nanoparticles and different ions and molecules in liquids [30]. The drop-coating method was used to make a MnO_2_-coated quartz sensor (Fig. S9). With different species of electrolytes adsorbing on the MnO_2_, the increased mass on the quartz sensor will lead to lower resonant frequencies (Δ*f*), which follows the equation Δ*f*/n ∝–Δ*m*. As shown in Fig. 3a, when the urea-free electrolyte (*x* = 0) passed through the quartz sensor, the Δ*f* descend indicated that OTf^−^ adsorbed on MnO_2_ which is consistent with its hydrophobic nature [3]. With the increased concentration of urea, the decreased Δ*f* indicates that urea tends to adsorb at the interface of MnO_2_, which may occupy the position of the OTf^−^ on the surface of the MnO_2_. This is further demonstrated by the Raman spectra of the mixed sludge of MnO_2_ and electrolytes. When in contact with MnO_2_, the solvation structure of electrolytes changed (Fig. 3b). The broad peak of Zn^2+^−OTf^−^ shifted from 1034.73 cm^−1^ to 1025.66 cm^−1^, which indicates that OTF^−^ could easily absorb MnO_2_ causing the bond length of Zn^2+^ and OTF^−^ to increase. The peak at ∼1025 cm^−1^ had blue-shifted to a higher wavenumber with the addition of urea, indicating that the adsorption intensity of OTF^−^ on the surface of MnO_2_ decreased. That's because, as a small polar organic molecule, urea tends to absorb on solid particles which may replace the adsorbed OTF^−^ on the interface of MnO_2_. What is noteworthy is the red-shifted peak when the concentration of urea exceeded 4 M, which can be attributed to the attraction of excess urea to the desorbed OTF^−^. As a comparison, electrolytes composed of 2 M ZnSO_4_ + *x* M Urea + 0.25 M MnSO_4_ are also used for the test (Fig. 3c). Although SO_4_^2−^ also adsorbs on MnO_2_, the change in urea adsorption mass with increasing urea concentration is not significant due to the difficulty of forming a strong hydrogen bond between it and urea (Fig. S10). Notice that when *x* = 6, the frequency of chips in Fig. 3c is significantly lower, which may be attributed to the fact that less hydrogen bonding between SO_4_^2−^ and urea takes place, which allows only excess urea to disrupt the adsorption equilibrium of the anion at the interface.

To further investigate the EDL of the cathode, the ζ potential of MnO_2_ particles in different electrolytes after dilution was tested (Fig. 3d). With the increased concentration of urea, the ζ potential showed an upward trend. It could attribute to the replacement of OTF^−^ by urea at the interface of MnO_2_ which increased the thickness of the EDL [31]. However, as the concentration of urea increased further (to 6 M), the ζ potential decreased instead. Although urea can replace OTF^−^, excessive additives compress the EDL of the cathode, thus decreasing the ζ potential. To summarize, the addition of urea significantly changed not only the solvation structure but also the situation of the cathode interface. This can be reflected in the change in entropy tested by the three-electrode system (Fig. S11). As the temperature changes, the voltage of the cathode will change too, the rate of voltage change with temperature is called the temperature coefficient (TC). TCs quantify this temperature dependency of the electrode voltage, which is proportional to the entropy change of the electrode interface [32]:


(1) \begin{eqnarray*}
\frac{{\partial E}}{{\partial T}} = - \frac{1}{{nF}}\frac{{\partial \Delta G}}{{\partial T}} = \frac{{\Delta S}}{{nF}},
\end{eqnarray*}

where *E* is the equilibrium cell voltage, *T* is the temperature, *n* is the number of electrons transferred in the reaction, *F* is Faraday's constant, and $\Delta G$ and $\Delta S$ are the Gibbs free energy and entropy change of the cathode reaction. As the urea concentration increases, the TC of the cathode interface goes up and so does the entropy (Fig. 3e). This is in line with the ‘entropy-driven’ principle for the adsorption of small organic molecules, demonstrating the substitution of urea for OTf^−^. Meanwhile, we carried out CV tests from 15°C to 45°C on cells containing different electrolytes to prove the stability of the interface (Fig. S12). It can be seen that there is a linear relationship between the peak position of the oxidation peaks and the temperature. Summarizing this trend in Fig. S13, when *x* = 4, the tendency for the oxidation voltage to change with temperature is minimal, which means that the *x* = 4 electrolyte has optimal interfacial stability and the interfacial composition is less susceptible to external environmental influences. Based on the above analysis of electrolyte and interface adsorption of the cathode, we can deduce the structure of the cathode EDL (Fig. 3f). After the addition of urea, the adsorption of urea at the interface crowds out the original OTf^−^ thus increasing the potential of the Stern layer.

### Analysis of deposition composition

Above we have analyzed ionic solvation structures and the EDL structure at the cathode interface. Using desolvation energy calculations, we analyzed the interaction between the solvation structure and the interfacial adsorbed substances. The energy barrier of the desolvation step is determined by the highest energy barrier of all the steps which is usually caused by the desolvation of the anion. In any electrolyte, the order of desolvation of Zn^2+^ and Mn^2+^ is OTf^−^ first (Table S2). It can be seen that the energy barriers of all the lowest energy barrier paths are not very different, so the second rate-determined steps are equally critical. Also, it is essential to consider the effect of the interface on the solvation structure [33]. In *x* = 4 electrolyte, considering the urea-rich environment at the cathode interface, desolvation of urea should be delayed. This, in turn, changes the second rate-determined step energy barrier of Zn^2+^ to −7.12 eV in *x* = 4 electrolyte (Fig. S14). Compared to the *x* = 0 electrolyte (Fig. S15), there is a significant drop in second rate-determined step energy barrier of Zn^2+^ (−7.12 eV to −4.52 eV), although the first is slightly higher (0.03 eV). Meanwhile, although the first rate-determined step energy barrier of Mn^2+^ rises to −10.72 eV, the second decreases to −4.49 eV compared to Zn^2+^ (Fig. S16). Therefore, we can assume that Zn^2+^ is restrained at the cathode interface.

**Figure 4.**
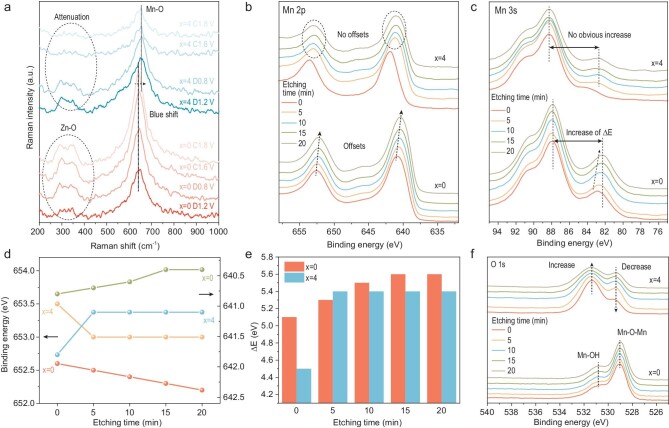
Analysis of the deposition composition. (a) *ex-situ* Raman spectra of cathodes at different charging or discharging states. XPS depth profile curves of etched cathode after 30 cycles at 0.2 A g^−1^ and charging to 1.8 V of (b) Mn 2p, (c) Mn 3s and (f) O 1s. (d) Specific variation in position of the Mn 2p peaks. (e) Specific variation in the spacing of the two peaks of Mn 3s.

As the general deposition composition of Zn*_x_*MnO(OH)*_y_*, the valence state of Mn increases with the decrease of *x* and increase of *y* [16,34]. The higher valence state of Mn means a satisfactory oxidation reaction depth during charging [35], and the low content of Zn in the deposition means the suppressed irreversible co-deposition of the product [36–38], thus increasing reaction capacity and stability. *Ex-situ* XPS patterns were used to characterize the composition of the cathode deposition after 100 cycles at a current density of 0.5 A g^−1^ (Fig. S17). The two broad peaks of Mn 2p was fitting into four peaks at ∼642.30 eV, 653.80 eV for low valance (+3) and 643.25 eV, 654.80 eV for high valance (+4) [39]. With the addition of urea, the valence states of Mn in the depositions increased obviously. Meanwhile, Raman spectra were also carried out on cathodes after charging and discharging to different voltages (Fig. 4a). The peaks at ∼300 cm^−1^ and ∼350 cm^−1^ imply the combination of Zn and O, while the peak at ∼640 cm^−1^ implies Mn and O [22,40]. The Mn-O peak blue-shifted with addition of urea to the electrolyte, which means the tighter Mn-O bond. The peak intensity of the Zn-O peak also decreases more obviously during charging, demonstrating that, in spite of the disappearance of zinc hydroxy sulphonate, the deposition is confirmed to contain less Zn. We also tested the XRD pattern of the cathode in different charging and discharging conditions (Fig. S18). When the electrolyte does not contain urea, ZnMn_3_O_7_ exists very obviously at the cathode during charging and discharging. On the contrary, in QEE, ZnMn_3_O_7_ at the cathode could hardly be seen during charging and discharging.

To further investigate cathode deposition, following low current cycling (0.1 A g^−1^, 30 cycles) cathodes were analyzed by the etched XPS depth profiles. For *x* = 0 electrolyte, the position of the Mn 2p peaks gradually shifted to lower energy with deeper etching but the position of the peaks remained essentially unchanged with increasing etch depth in the *x* = 4 electrolyte (Fig. 4b). For the Mn 3 s peaks, the binding energy difference ΔE increased with increasing etch depth in *x* = 0 electrolyte but barely increased in *x* = 4 electrolyte (Fig. 4c). The position of the Mn 2p peaks shifted from 640.8 and 652.6 eV to 640.4 and 652.2 eV, respectively, as the depth of etching increases in *x* = 0 electrolyte, implying a gradual decrease in the valence state of Mn in the deposition. On the contrary, the position of the peaks remained essentially unchanged at 641.1 and 653 eV with increasing etch depth in the *x* = 4 electrolyte, implying no significant change in the valence state of Mn (Fig. 4d). It is noteworthy that the Mn in the cathode of the *x* = 4 electrolyte shows a significant valence rise to 641.8 and 653.5 eV at the end of the deposition. This is because, the sharp rise in voltage towards the end of the charging process creates an excessively strong electric field that repels the unreacted Zn^2+^ near the cathode. Meanwhile, the Zn^2+^ at the cathode interface in the *x* = 0 electrolyte is continuously involved in the reaction, and such constant reaction implies a smoothly changing voltage plateau without sudden increase. Figure 4e further illustrates this valence change through the XPS curves of the Mn 3 s. The energy difference (ΔE) between the two peaks of Mn 3 s responds to the valence state of Mn [41]. The ΔE of *x* = 0 electrolyte changed from 5.1 eV to 5.6 eV with increasing etch depth while the ΔE of *x* = 4 electrolyte basically maintained at 5.4 eV (Fig. 4e), further validating the unchanged valence. Also, the XPS curves of the O 1s orbital give further insight into the existing form of O in the deposition (Fig. 4f). The lower energy peak at ∼529 eV represents the Mn−O−Mn binding form and the higher energy peak at ∼531 eV represents the Mn−OH binding form [42]. Compared to the *x* = 0 electrolyte, the *x* = 4 electrolyte showed a significant increase in the Mn−OH bonds, while the peak of Mn−O−Mn bonds decreased significantly. This means that in the *x* = 4 electrolyte, O would be more inclined to be present in the combined form of Mn−OH. Therefore, the *y* value in Zn*_x_*MnO(OH)*_y_* of the *x* = 4 electrolyte will increase, side-by-side with the increase in the valence of Mn. Mass transfer modulation by QEE ultimately leads to an increase in the valence of Mn and the decrease in the content of Zn in the deposition, promoting high capacity and reversibility.

### Analysis of the initial and the stable-cycle kinetics

Theoretically, changes in EDL potential can affect the oxidation kinetics at interface. Ideally, according to the Butler–Volmer equation, the rate of oxidation reaction at the electrode can be expressed as follow [43]:


(2) \begin{eqnarray*}
{i}^o = FAC\left( {x,\ t} \right){\mathrm{exp}}\left[ {\alpha \frac{F}{{RT}}\left( {E - {E}^0} \right)} \right],
\end{eqnarray*}

where ${i}^o$ represents the rate of oxidation reaction, *F* and *R* are Faraday's constant and molar gas constant, *A* is reaction area, $C( {x,\ t} ){\mathrm{\ }}$presents concentration of ions at distance $x{\mathrm{\ }}$from the electrode surface and at time *t*, $\alpha $ is transfer coefficient, *E* and ${E}^0$ are actual electrode potential and equilibrium electrode potential, respectively. After the consideration of modification of electrode potential by adsorbents on the electrode, the equation will be amended as follows [43]:


(3) \begin{eqnarray*}
{i}^o &=& FAC\left( {\infty ,\ t} \right)\exp \left( {\frac{{ - {z}_ie{\varphi }_2}}{{kT}}} \right)\nonumber\\
&&\times {\mathrm{exp}}\left[ {\alpha \frac{F}{{RT}}\left( {E - {\varphi }_2 - {E}^0} \right)} \right],
\end{eqnarray*}

where $C( {\infty ,\ t} )$ represents concentration of ions at zero potential, ${\varphi }_2$ represents the potential of the Stern plane. The overall effect of this on electrode kinetics is known as ${\varphi }_2$ effect [43]. It can be seen that the rate of the interface oxidation reaction decreases as ${\varphi }_2$ increase, which is detrimental to the kinetics.

Therefore, it can be argued that the initial kinetics of the QEE at the cathode are unfavorable. However, analysis of the kinetics should focus on the phase of stable dissolution deposition of the cathode. However, from a depositional morphological point of view, the situation has changed.

**Figure 5.**
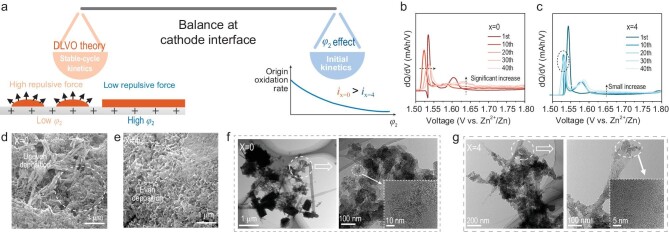
Electrochemical phenomenon and deposition morphology during cycling (a) Schematic diagram of the balance problem at the cathode interface. (b and c) dQ/dV curves of cells cycled in (b) x = 0 electrolytes and (c) x = 4 electrolytes. (d and e) *ex-situ* SEM images of cathode surface morphology after charging to 1.8 V and (f and g) *ex-situ* TEM images of cathode morphology after charging to 1.8 V.

In QEE, the OTf^−^ at the interface was replaced, which led to a rise in the potential of the Stern layer. According to DLVO theory, the higher the potential *φ*, the greater the repulsive force *V_R_* between substances [44]. The greater the repulsive force *V_R_*, the less likely it is that substances will agglomerate. Thus, higher interfacial potentials in the QEE can alleviate deposition agglomeration, resulting in even deposition during a cycle. More even deposition allows for better contact between the active substance and the conductive carbon, resulting in stable and efficient charge transfer. Therefore, we need to explore the balance between initial kinetics and stable-cycle kinetics (Fig. 5a).

The dQ/dV curves of different electrolytes were measured (Fig. 5b and c, and Fig. S19), exhibiting two plateaus in the charge process (Fig. S20). It is worth noting that in *x* = 0 electrolyte, the potential of the first plateau decreases initially and then gradually increases (Fig. 5b). However, in *x* = 4 electrolyte, although the potential of the first charging plateau increases slightly on the first cycle, the potential of the first plateau gradually decreases as the cell is cycled and does not significantly increase again (Fig. 5c). Compared to the first plateau, there are clear differences in the second plateau. When the electrolyte with urea is charged, the second plateau almost disappears during the first charge process. However, in subsequent cycles, the second plateau reappears and is lower than the plateau voltage of the urea-free electrolyte during the first charge process. At the same time, the plateau of the urea-free electrolyte at the higher voltage appears to rise significantly as the cycle progresses, indicating a very serious polarization. In summary, it can be analyzed that the kinetics of the urea-free electrolyte is superior to that of the QEE electrolyte at the first charge turn under the condition of the same cathode interface morphology. However, as the cycle progresses, the kinetics of the urea-added electrolyte is again superior to that of the urea-free electrolyte.

This is most likely due to the change in electrode morphology during cycling. SEM and TEM of the cathode interface were carried out to investigate further. As shown in Fig. 5d, the deposition morphology is very uneven and shows significant agglomeration without the addition of urea. In contrast, the deposited morphology became homogeneous when the urea concentration was 4 M (Fig. 5e). This phenomenon matched with the DLVO theory [45]. The TEM test further demonstrates this phenomenon. Significant agglomeration of stratified depositions can be seen in Fig. 5f, while this is not the case in Fig. 5g. In addition, the *x* = 0 electrolyte causes depositions to agglomerate on the conductive carbon (Fig. S21), while in the *x* = 4 electrolyte depositions are mostly present as a single layer on the conductive carbon (Fig. S22) indicating better contact of deposition with conductive carbon.

Adsorption of urea at the cathode interface raises the potential of the Stern layer. Despite the deterioration of the QEE kinetics at the beginning of the cycle, it allows for a more even deposition, and a more even deposition allows for better kinetics of the cathode during stable cycling. Therefore, when regulating the kinetics, we should pay attention to the balance between the initial kinetics and the kinetics during the stable cycle. More attention should be paid to the kinetics during the stable cycle than to the initial kinetics. The initial interfacial reaction energy barrier is not the main factor affecting the cycling performance, and the good reaction kinetics induced by interfacial deposition during the cycling process is more conducive to the stable cycling of the battery. It also demonstrates that the optimization of charge transfer by QEE is sustainable.

### Electrochemical performance

**Figure 6.**
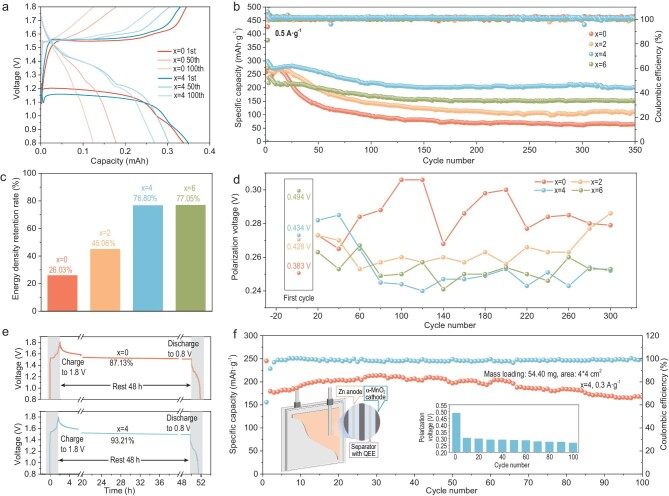
Electrochemical performance. (a) Galvanostatic charge and discharge profiles of cells in *x* = 0 and *x* = 4 electrolytes at 0.5 A g^–1^. (b) Cycle performances at 0.5 A g^–1^. (c) Energy density retention rate of electrolytes after 350 cycles. (d) Polarization voltage of the cells at different number of cycles. (e) Self-discharge curves of *x* = 0 and *x* = 4 electrolytes. (f) Cycle performances of soft-pack battery at 0.3 A g^–1^.

From the above, it can be seen that the QEE regulates the mass transfer and charge transfer of ions at the cathode interface, thus changing the deposition valence and the deposition morphology of the cathode, which directly affects the performance of the cells. The cells with different electrolytes were tested at 0.5 A g^−1^. First, we analyzed the charging and discharging behavior of the battery in (Fig 6a). It can be seen that within 100 cycles, the *x* = 0 electrolyte had become significantly polarized and visible plateaus had almost disappeared while *x* = 4 electrolyte maintained steady plateaus (Fig. 6b). In the first few cycles, cells with low urea concentration (0 M, 2 M) electrolytes had better performance, which was matched with their kinetics. The specific capacity could reach ∼300 mAh g^−1^. However, as the reaction went on, the performance of cells with low urea-concentration electrolytes deteriorated severely. In contrast, when the concentration of urea reached 4 M, the specific capacity of the cell did not decay as dramatically as the cell cycle. With further increases in urea concentration, the cell still had good cycle stability, but the capacity began to drop. This might be due to the fact that too high a concentration of urea leaves too little water in the electrolyte, which was not conducive to the dissolution and deposition of the cathode. The coulombic efficiency remains close to 100% throughout the cycle, indicating that the capacity decay is not due to irreversible Mn dissolution, but rather to the degraded kinetics causing by uneven deposition morphology and irreversible Mn deposition. After 350 cycles at 0.5 A g^−1^, the *x* = 4 electrolyte maintains an energy density of 300 Wh kg^−1^ (Fig. S23). Also, compared to the low energy density retention rate of the *x* = 0 electrolyte (26.03%), the *x* = 4 electrolyte can achieve a retention of 76.80% (Fig. 6c) which increased by 174% after 350 cycles, further demonstrating QEE’s improvement in the stability of ZMBs. As a comparison, we replaced Zn(OTf)_2_ in the electrolyte with the same concentration of ZnSO_4_ and cycled it using the same current conditions. It can be noted that the cell with ZnSO_4_ produced a significant capacity degradation (Fig. S24), which indicates that it is not just the urea itself that changes the performance of the cell, but the interaction it produces with OTf^−^. The rate performance of cells from 0.2 mAh g^−1^ to 1.4 mAh g^−1^ was carried out (Fig. S25). As the concentration of urea increases, the capacity decays less due to increased current (Fig. S26). This suggests that the addition of urea has improved the overall cell kinetics, which further demonstrates that the effect of urea on kinetics should not be limited to the early stages of the reaction, but should take into account the effect of changes in morphology on kinetics as the reaction progresses.

Figure 6d shows the polarization voltage of the cells at a different number of cycles by calculating the voltage difference between charging and discharging (both at half capacity). In the first cycle, the polarization voltage increases with the increasing urea concentration which, once again, demonstrates that the addition of urea is detrimental to the initial kinetics of the reaction. However, as the reaction proceeds, the polarization voltage for the *x* = 0 electrolyte was much greater than for other electrolytes and fluctuates significantly. When the urea concentration in the electrolyte continues to rise to 4 M and above, the polarization voltage remains stable and at a low value as the number of cell cycles increases. This is further evidence that the effect of urea on the kinetics changes from unfavorable to optimal as the cycle progresses. The effect of changes in cathode morphology and kinetics on the battery can also be seen in the self-discharge curve (Fig. 6e and Fig. S27). After 5 cycles of reaction at 0.1 A g^−1^ current, the battery was charged to 1.8 V, then rested for 48 h and discharged to 0.8 V. There is a significant increase in capacity retention after the addition of urea (from 87.13% to >90%), which means that the addition of urea reduces irreversible reactions at the battery interface.

To further explore the commercial potential of QEE, we then assembled a three-layer stacked soft-pack battery to test the industrial potential of QEE. The battery can be cycled for 100 turns at 0.3 A g^−1^ and maintain a specific capacity of ∼200 mAh g^−1^ (Fig. 6f). At the same time, the charge/discharge curves still retained a similar shape (Fig. S28), further demonstrating the stability of the soft-pack battery. It is noteworthy that the polarization voltage decreases as the reaction progresses after the first cycle and maintains a steady value (insert in Fig. 6f), demonstrating the stable and superior kinetics of the soft-pack battery during the cycle.

## CONCLUSION

This work developed the feasibility of quasi-eutectic electrolytes (QEEs) in zinc–manganese batteries, in which the optimization of ion solvation structure and Stern layer composition modulates the mass transfer and charge transfer at the cathode interface. In detail, the OTf^−^ enters the solvation structure of the cation and changes the overall charge of the solvation structure according to the hydrogen bonds. At the same time, due to differences in polarization rates caused by differences in the number of electrons in the outermost orbitals, urea tends to enter the solvation structure of Zn^2+^ but hardly enters the solvation structure of Mn^2+^. At the MnO_2_ cathode interface, urea displaces the OTf^−^ adsorbed on the cathode, thereby increasing the Stern layer potential and further improving the repulsive force of depositions which eventually optimized the interfacial charge transfer kinetics in subsequent cycles. Additionally, the differences in the solvation structures of Zn^2+^ and Mn^2+^ make the two behave differently at the positive end of urea adsorption, with Zn^2+^ being limited by urea and Mn^2+^ not. This leads to an elevated valence of Mn in the deposition product, resulting in a higher capacity and better reversibility. As a result, QEE maintained a stable specific capacity of 250 mAh g^−1^ at 0.5 A g^−1^ after 350 cycles in ZMBs. Furthermore, the multi-stacked soft-pack battery with a cathodic mass load of 54.4 mg maintained a stable specific capacity of 200 mAh g^−1^ for 100 cycles, demonstrating its commercial potential. This work provides the feasibility of adapting lean-water QEE to the stable aqueous ZMBs.

## MATERIALS AND METHODS

Detailed materials and methods are available in the Supplementary data.

## Supplementary Material

nwad220_Supplemental_File
