# Supplementary material for: Reconstructing interfacial manganese deposition for durable aqueous zinc–manganese batteries
Source: Natl Sci Rev. 2023 Aug 16;10(10):nwad220. doi: 10.1093/nsr/nwad220 (PMC10484177; doi:10.1093/nsr/nwad220)
Supplement: nwad220_Supplemental_File [file nwad220_supplemental_file.pdf]

Supporting Information

Reconstructing interfacial manganese deposition for durable aqueous zinc-manganese batteries

Yida Hu,[1,†] Zhexuan Liu,[1,†] Lanyan Li,[2] Shan Guo,[1] Xuefang Xie,[3] Zhigao Luo,[4] Guozhao Fang,[1,*] Shuquan Liang[1,*]

[1]*School of Materials Science and Engineering, Key Laboratory of Electronic Packaging and Advanced Functional Materials of Hunan Province, Central South University, Changsha 410083, P.R. China.*

[2]*School of Science, Hunan University of Technology and Business, Changsha, 410205, China*

[3]*College of Physical Science and Technology, Xinjiang University, Urumqi 830046, China.*

[4]*College of Chemistry, Xiangtan University, Xiangtan 411105, P.R. China.*

†These authors contributed equally to this work.

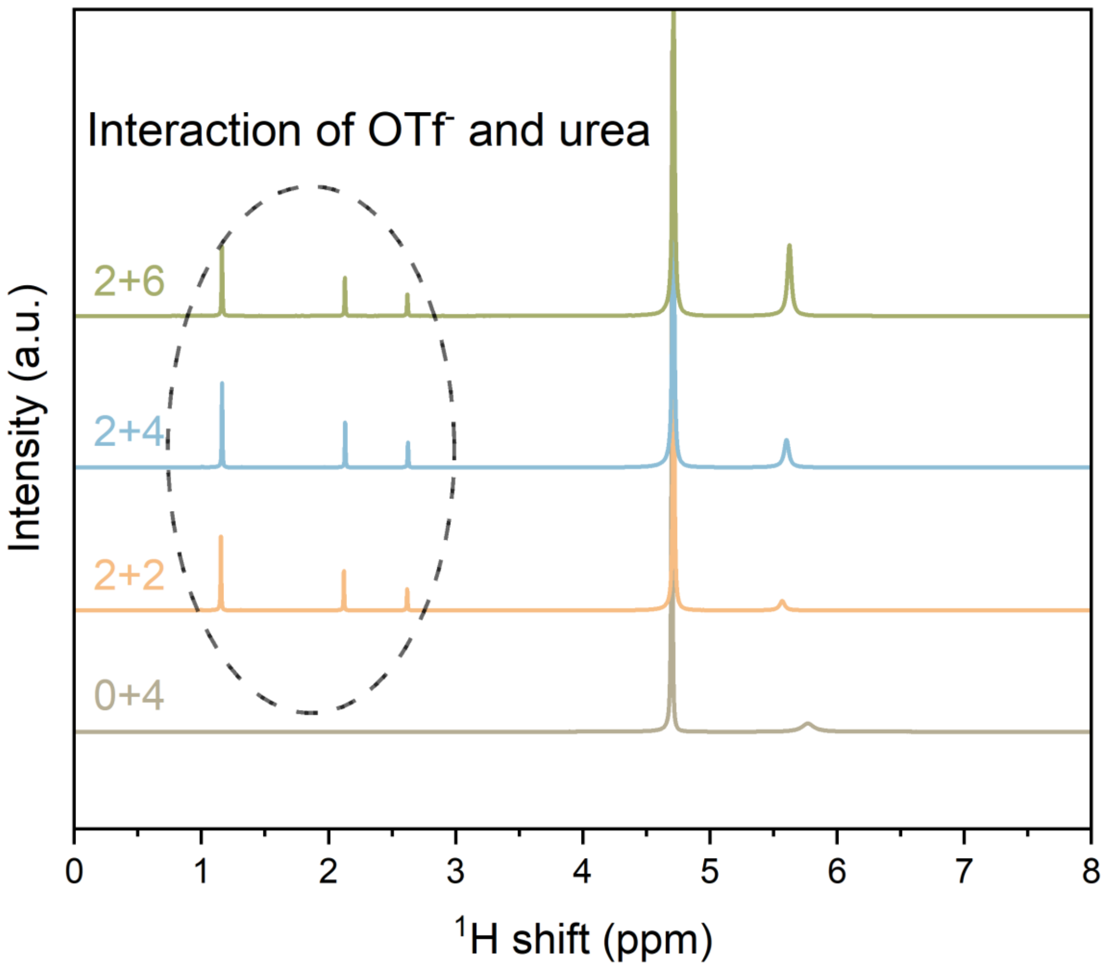

Figure S1 ^{1}H NMR of each electrolyte.

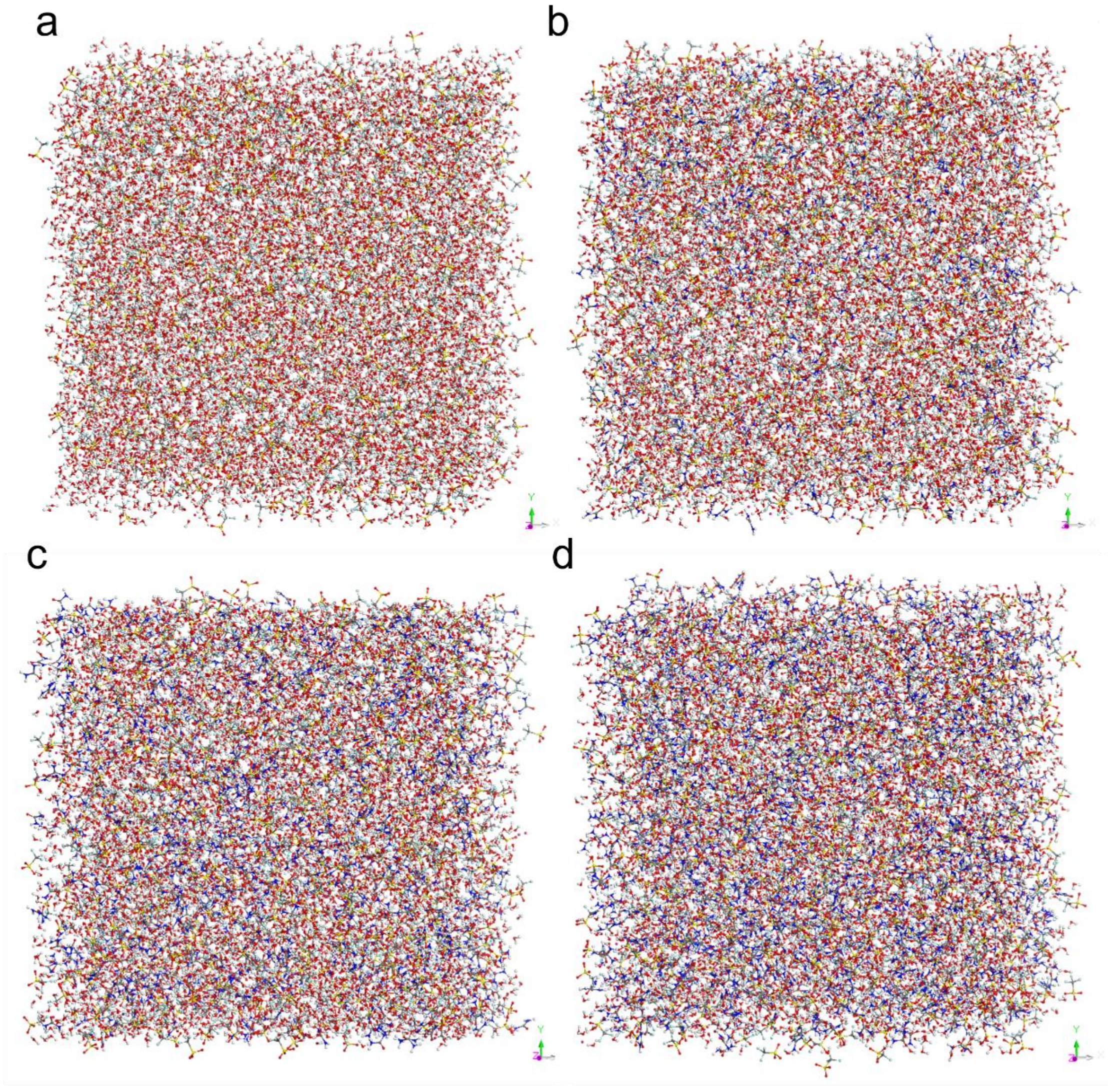

Figure S2 Molecular dynamics (MD) calculations boxes of each electrolyte. (a) x=0. (b) x=2. (c) x=4. (d) x=6.

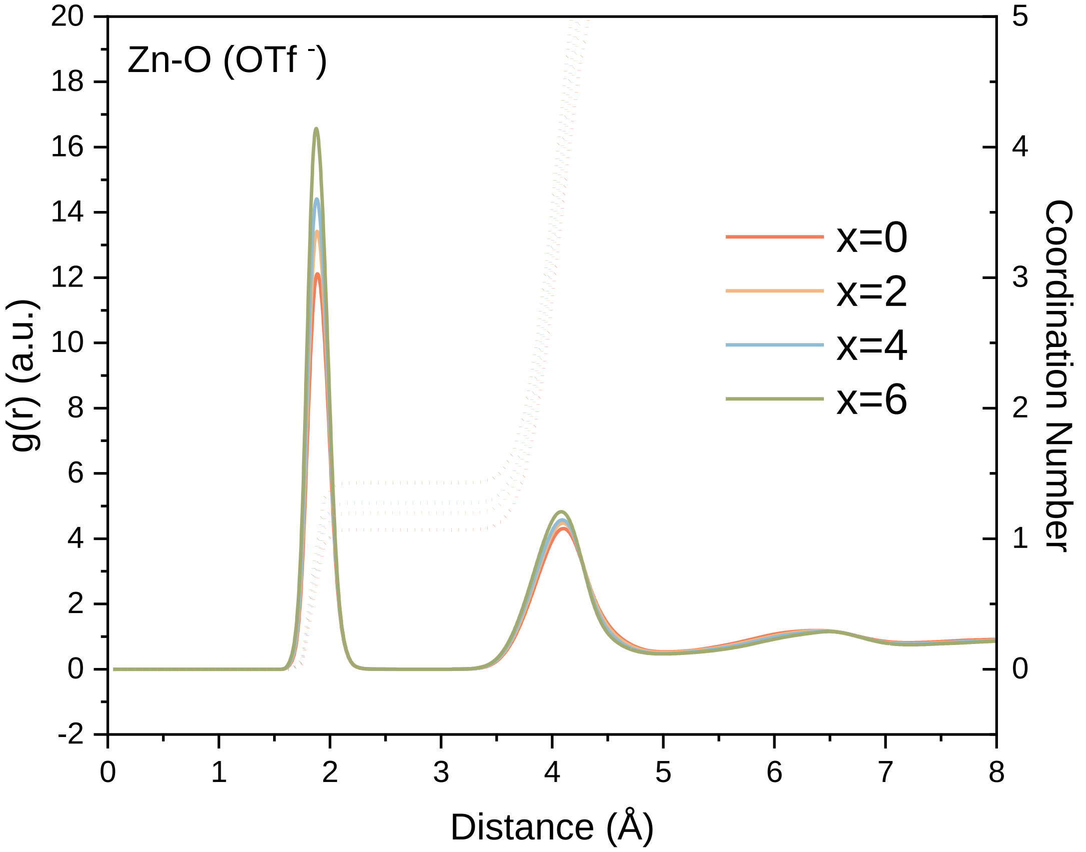

Figure S3 Radial distribution function (RDF) for Zn-O(OTf^-)

.

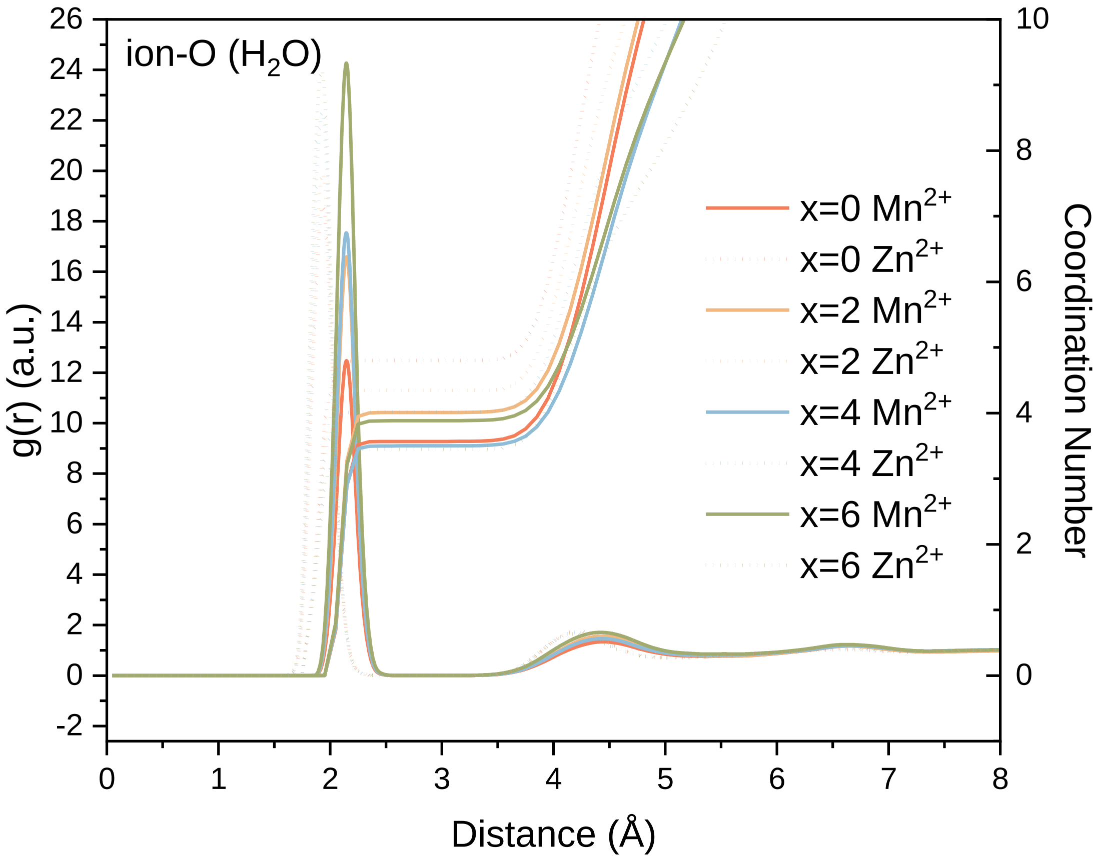

Figure S4 Radial distribution function (RDF) for Zn-(O)H_2O and Mn-(O)H_2O pairs.

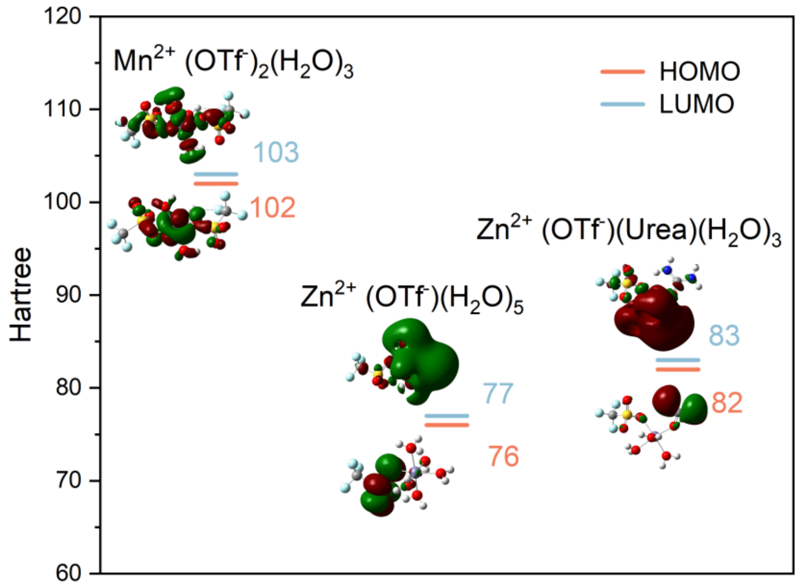

Figure S5 LUMO and HOMO of different solvation structures.

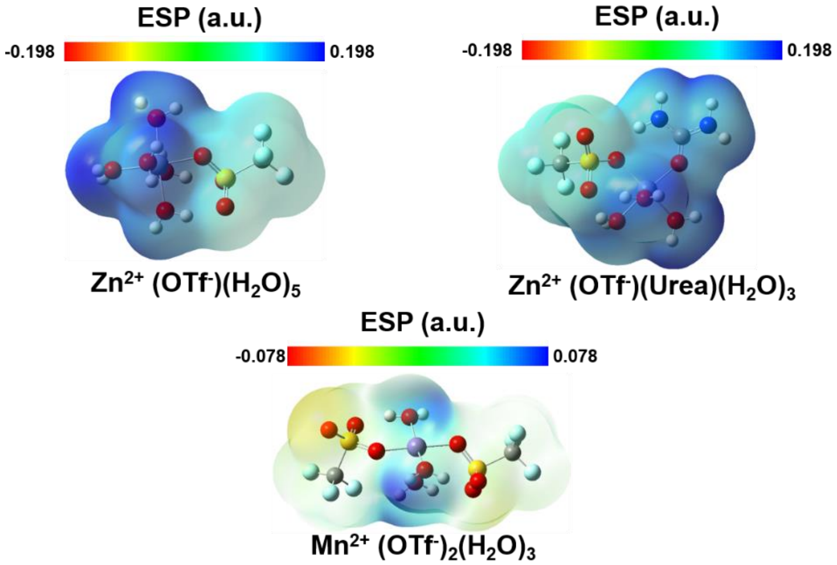

Figure S6 Electrostatic potential for different solvation structures.

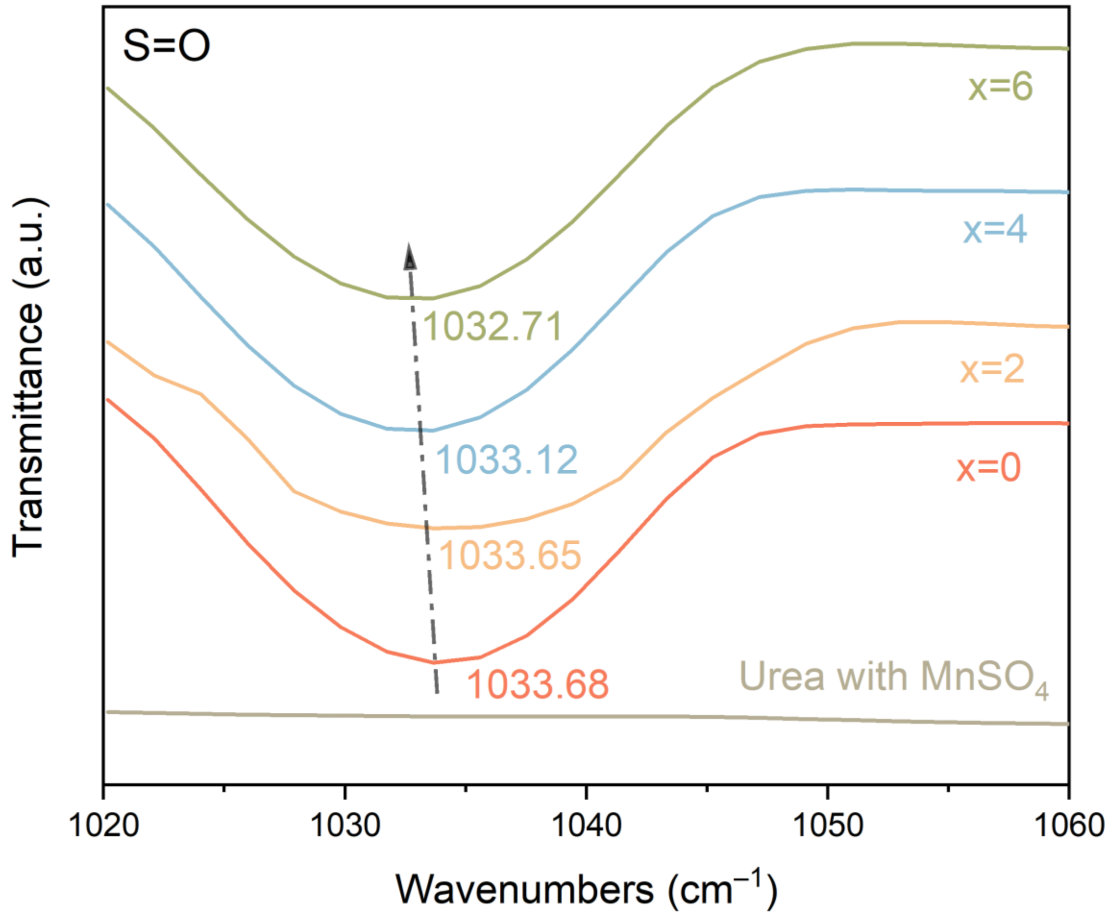

Figure S7 FTIR spectra of electrolytes at from 1020 cm^{-1} to 1060 cm^{-1}.

.

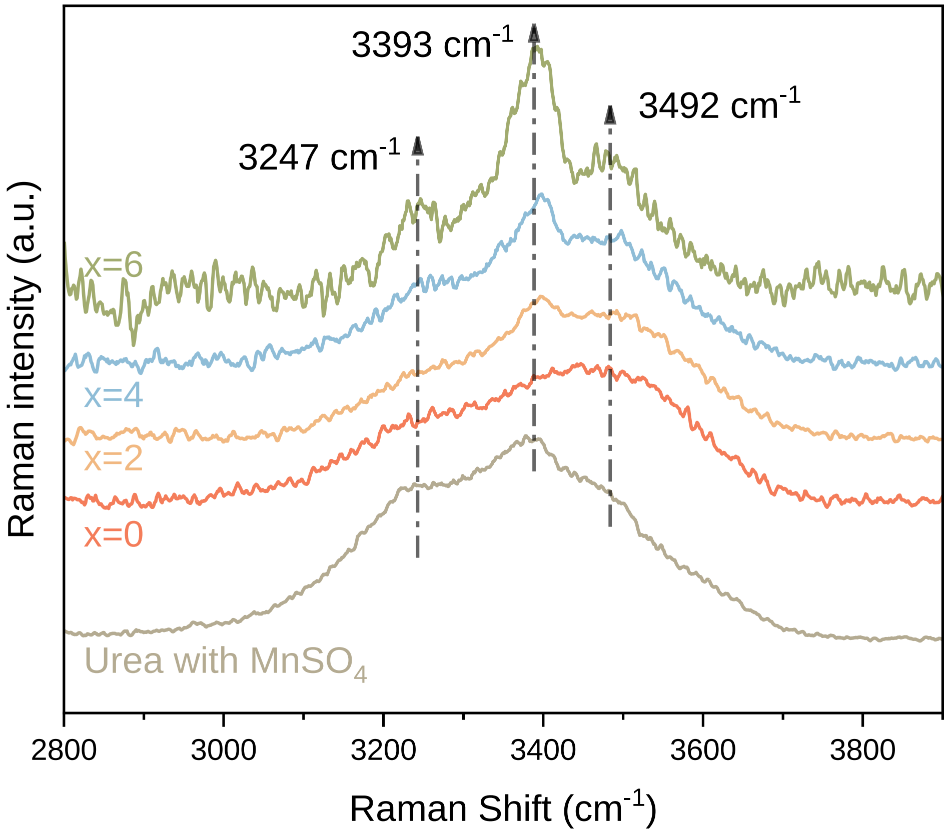

Figure S8 Raman spectra of electrolytes at 2800–3900 cm^{-1} representing the stretching vibration of −NH_2.

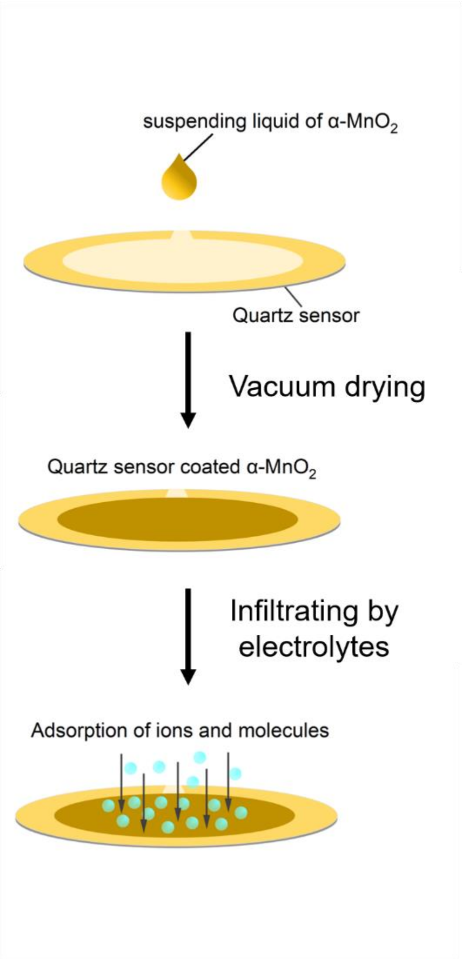

Figure S9 Diagram of the drop-coating method.

10 mg of α-MnO_2 was ground and added to 200 ml of deionized water and sonicated for 10 minutes to a suspension. 100 μL of the suspension was placed on the chip and dried in a vacuum drying oven at 60 °C for 2 h.

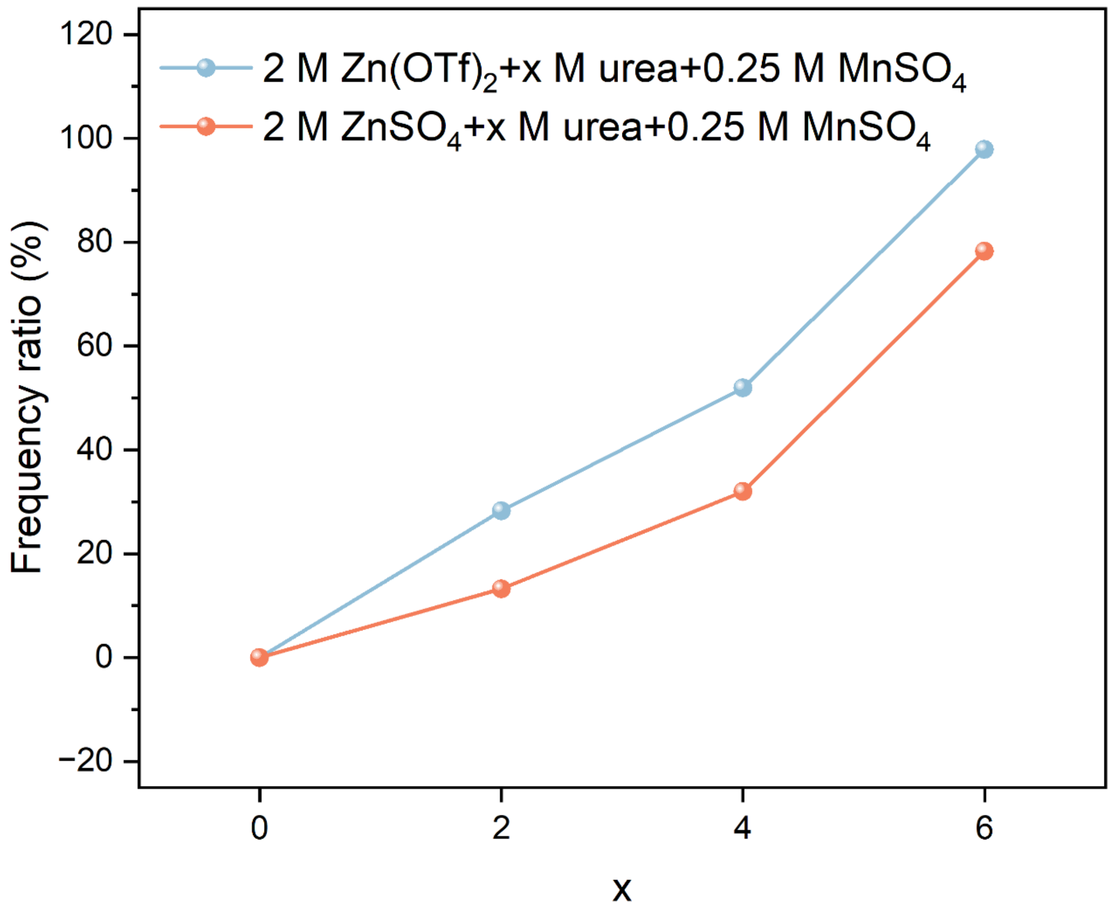

Figure S10 The change of frequency ratio in Fig. 3a and Fig. 3c.

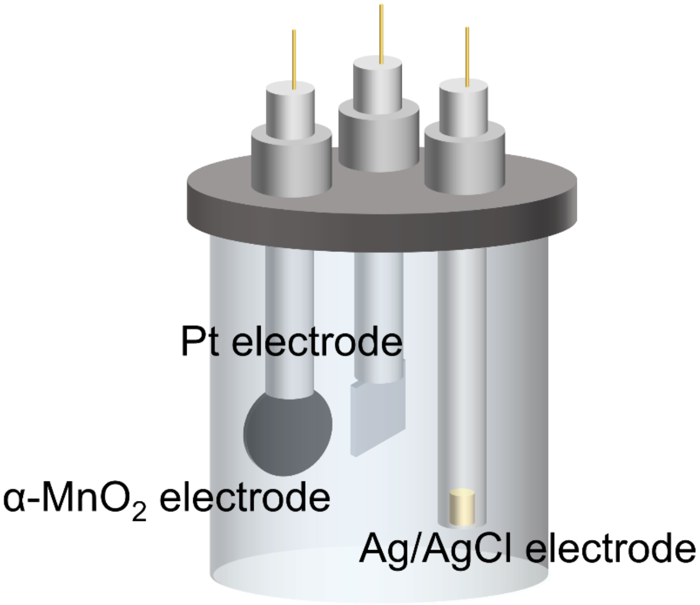

Figure S11 Schematic of the three-electrode cell. Ag/AgCl electrode was reference electrode, Pt electrode was counter electrode and α-MnO_2 electrode was working electrode.

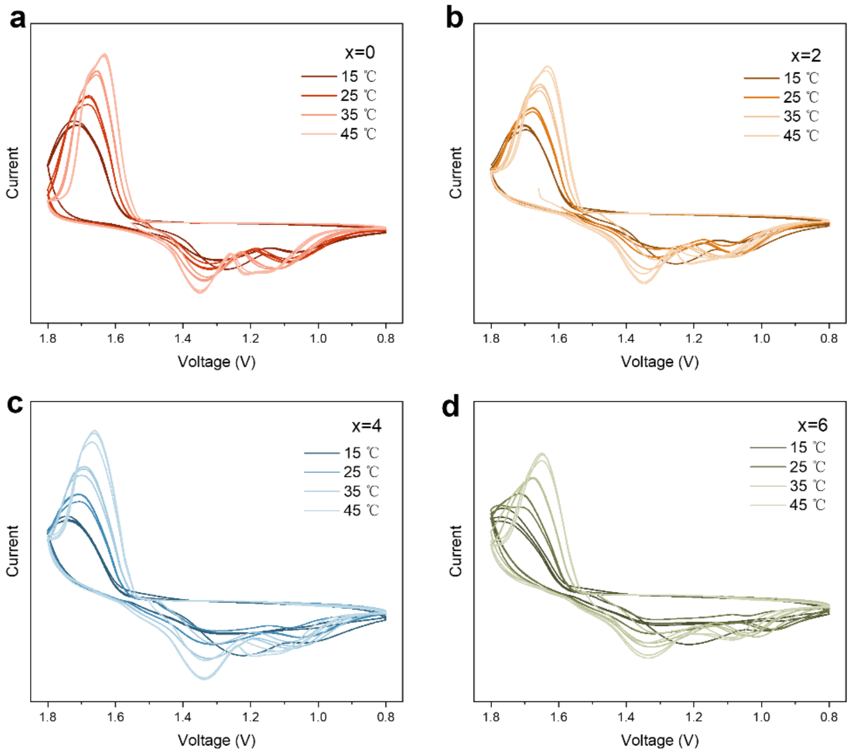

Figure S12 CV curves at different temperature for different electrolytes at scan rate 1 mV s^{-1}. (a) x=0. (b) x=2. (c) x=4. (d) x=6.

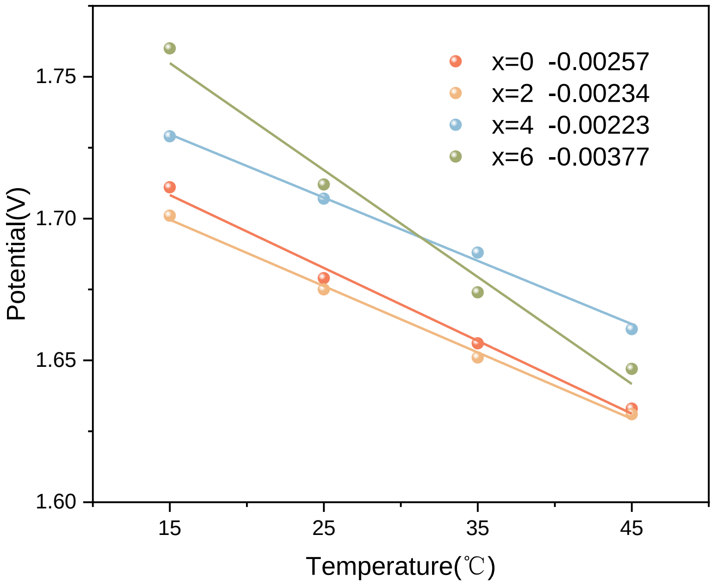

Figure S13 Oxidation voltages for the different CV curves in Figure R1 and their fits.

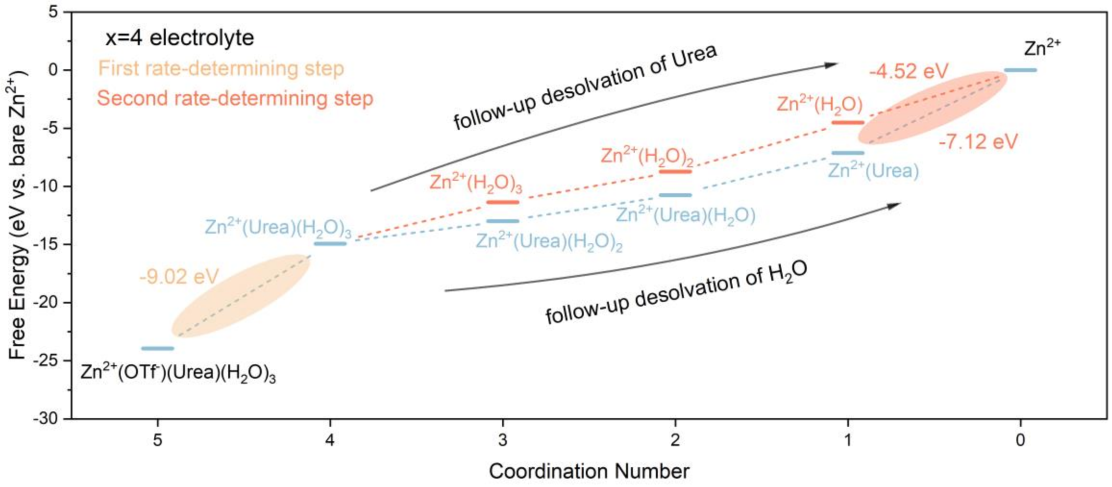

Figure S14 Two pathes for the desolvation of Zn^{2+} in x=4 electrolyte (desolvation of OTf^- first).

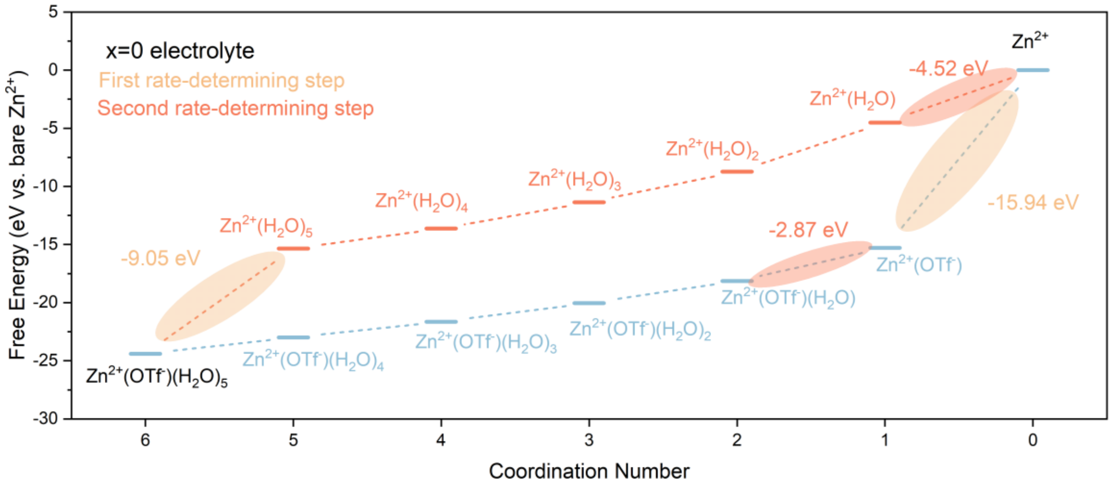

Figure S15 Two pathes for the desolvation of Zn^{2+} in x=0 electrolyte.

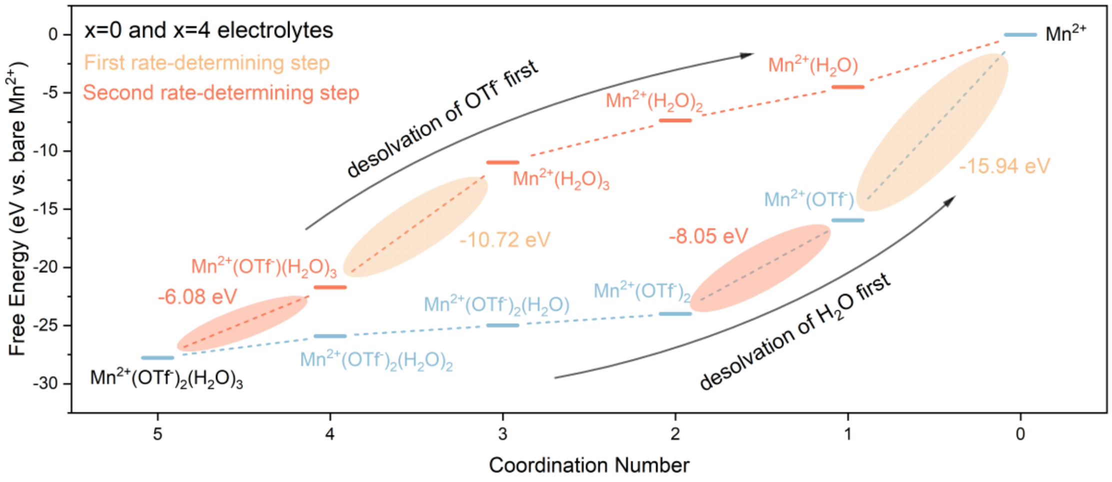

Figure S16 Two pathes for the desolvation of Mn^{2+} in both x=0 and x=4 electrolyte.

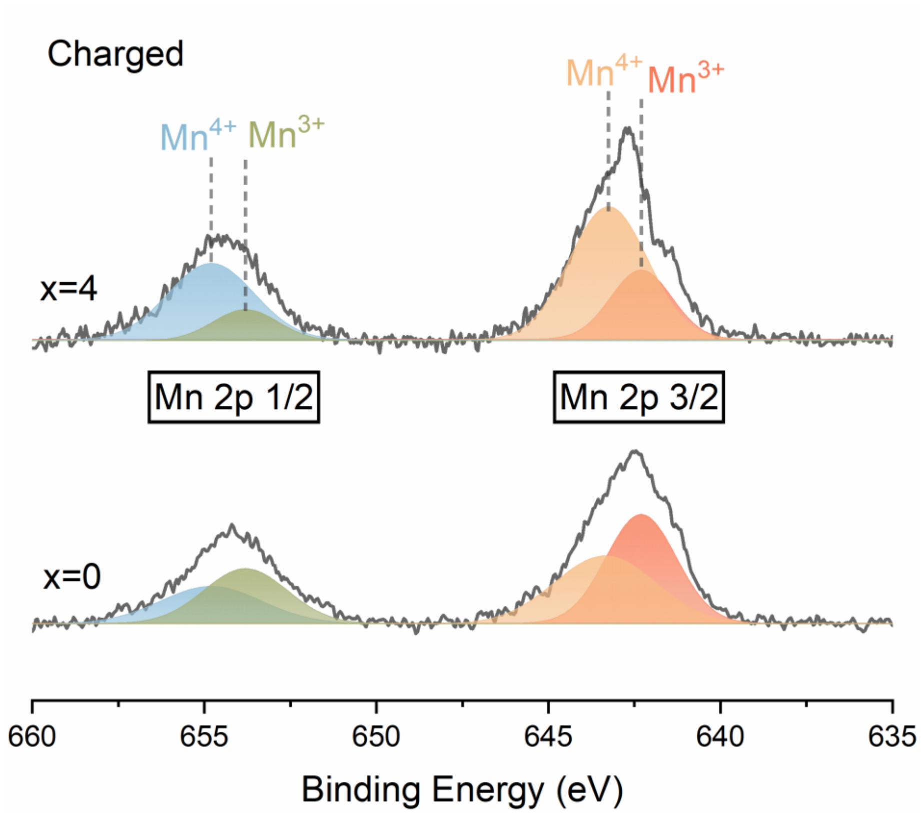

Figure S17 *Ex-situ* XPS curves of cathode surface after 100 cycles at 0.5 $A \cdot g^{-1}$ and charging to 1.8 V.

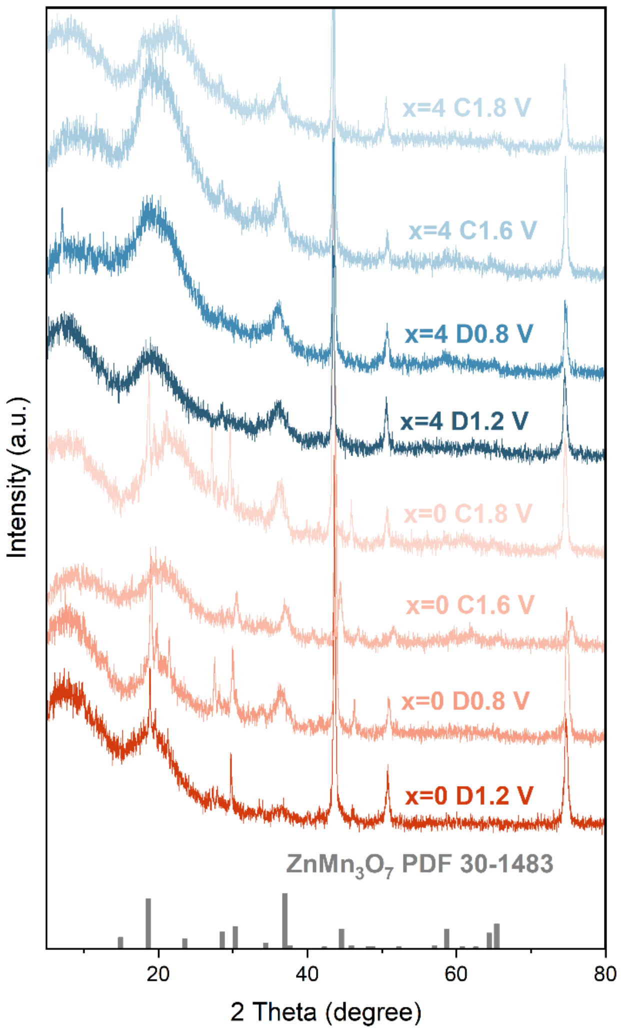

Figure S18 XRD pattern of cathode under different charging and discharging conditions after 100 cycles at 0.5 A g^{-1}.

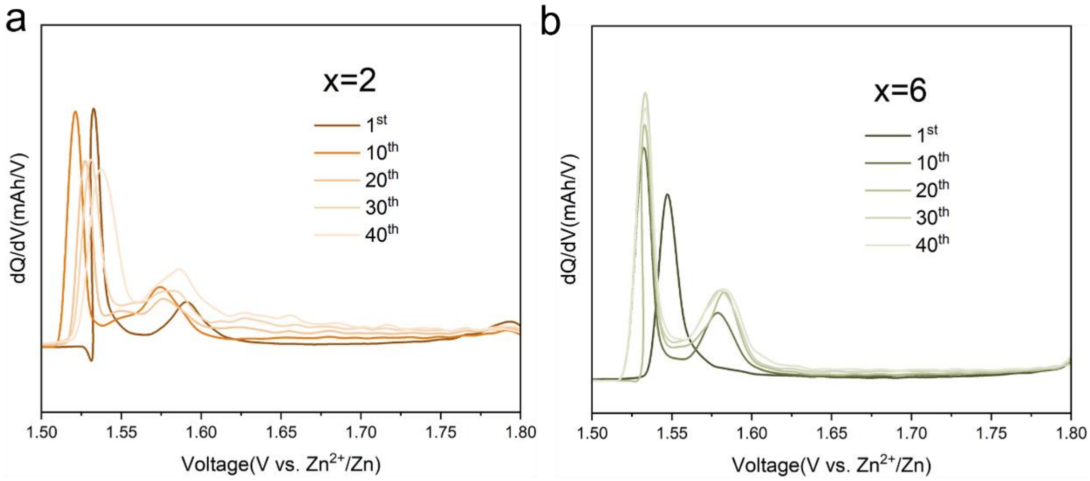

Figure S19 dQ/dV curves of different electrolytes. (a) x=2, (b) x=6.

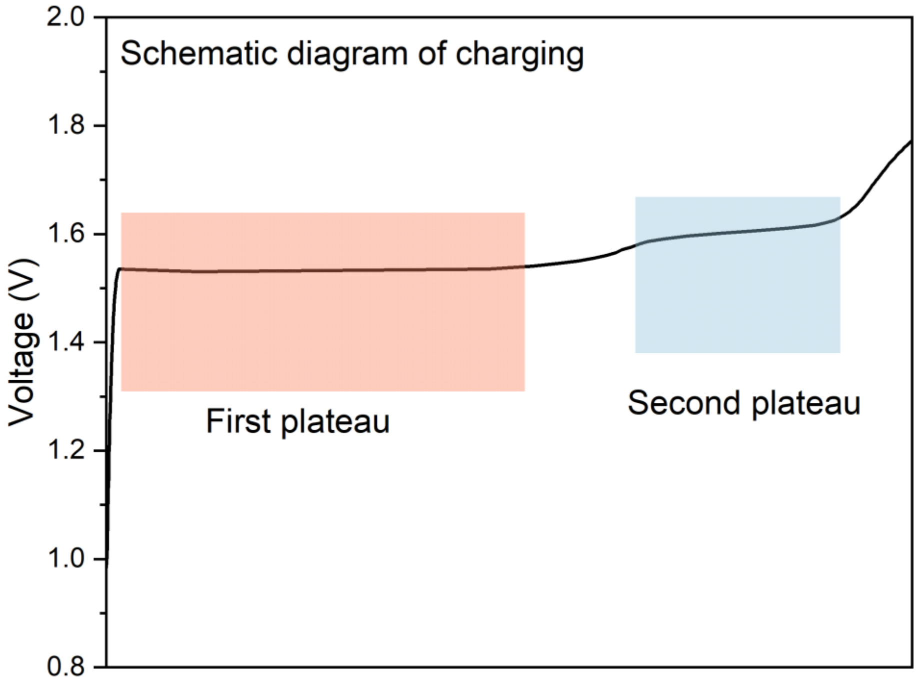

Figure S20 Schematic diagram of charging. The red area indicated the first platform and the blue area indicated the second platform.

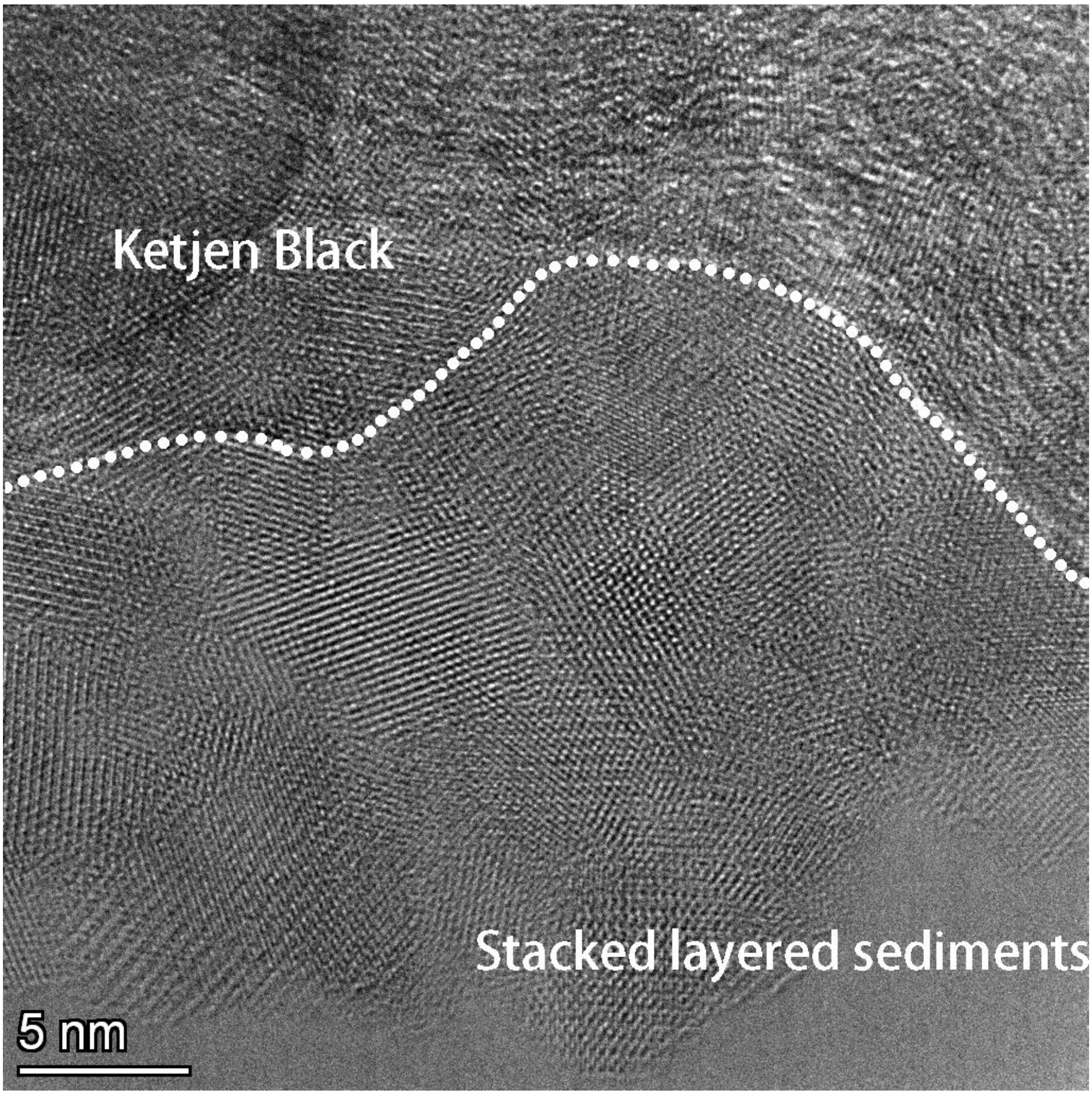

Figure S21 TEM of x=0 electrolyte charged to 1.8 V.

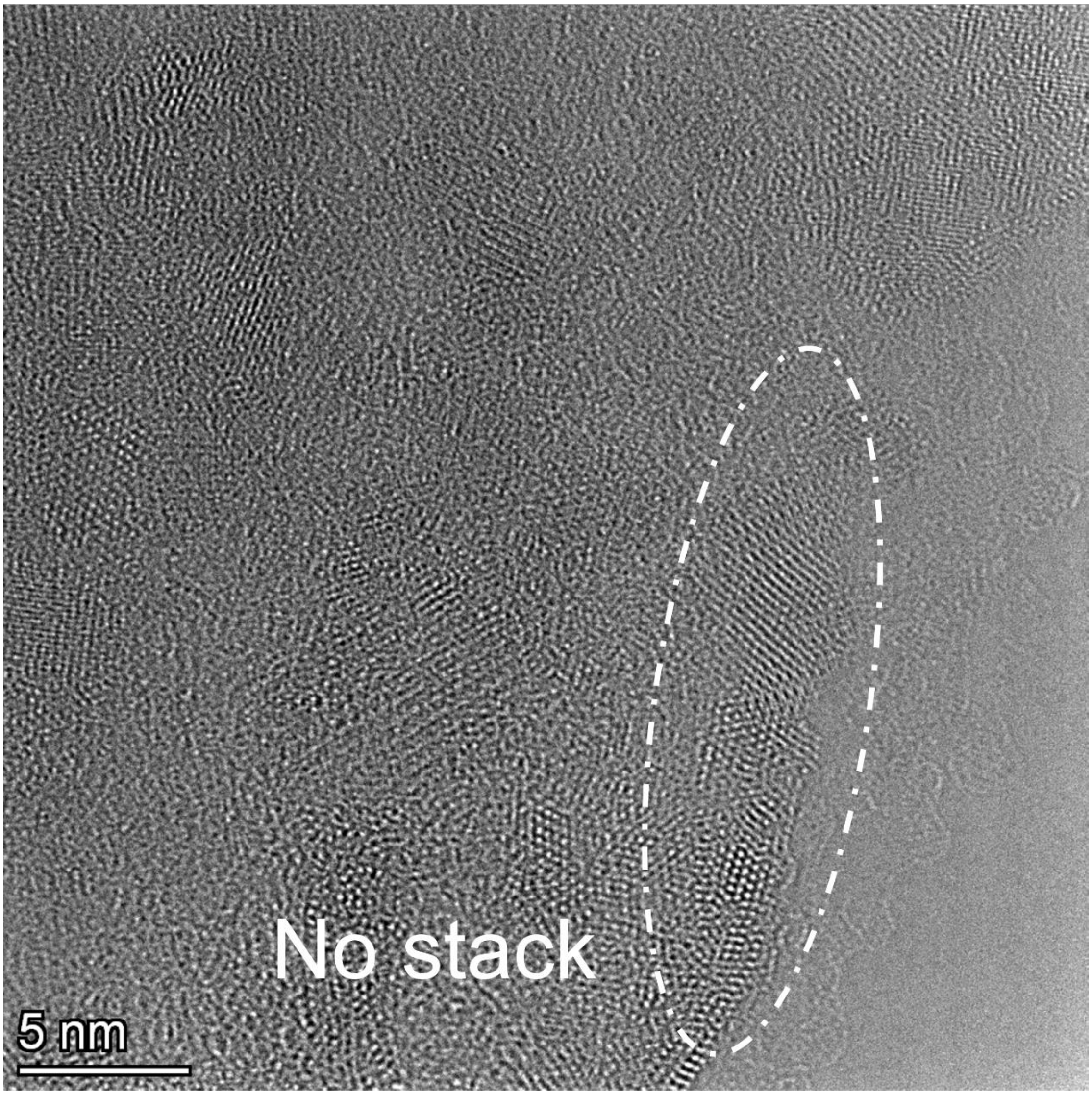

Figure S22 TEM of x=4 electrolyte charged to 1.8 V.

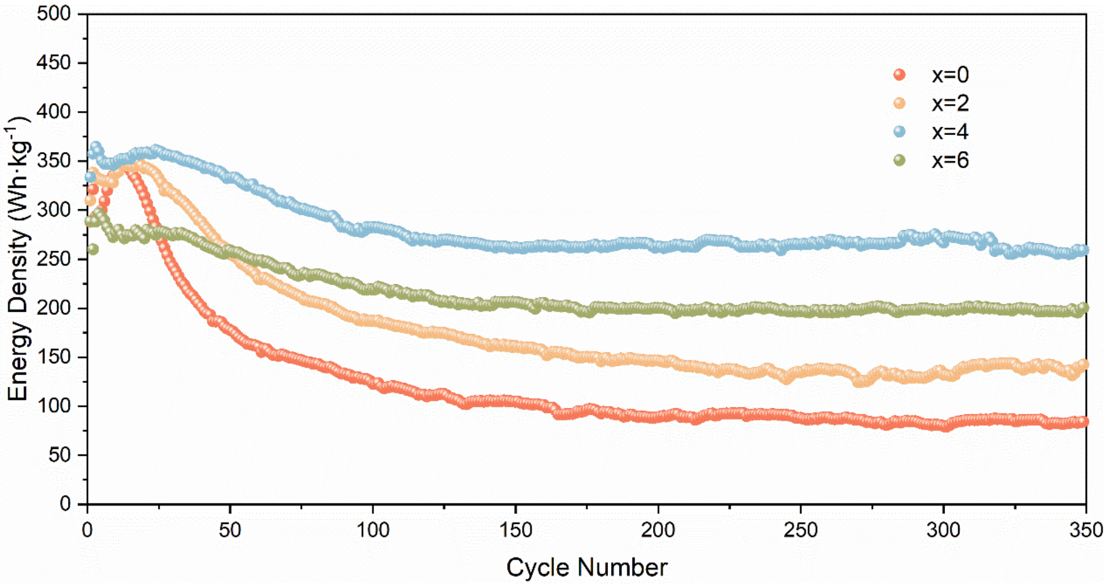

Figure S23 The energy density (mass of active substance) of cells at 0.5 A·g^{-1}

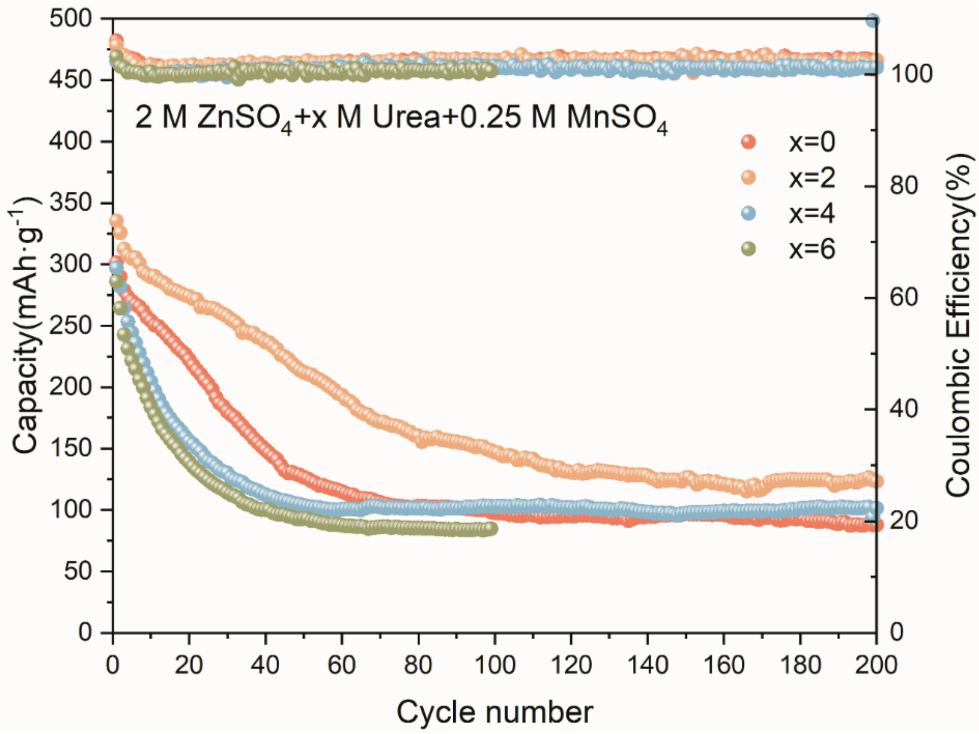

Figure S24 Full-cell performance of cells with $ZnSO_4$ at 0.5 A·g^{-1}.

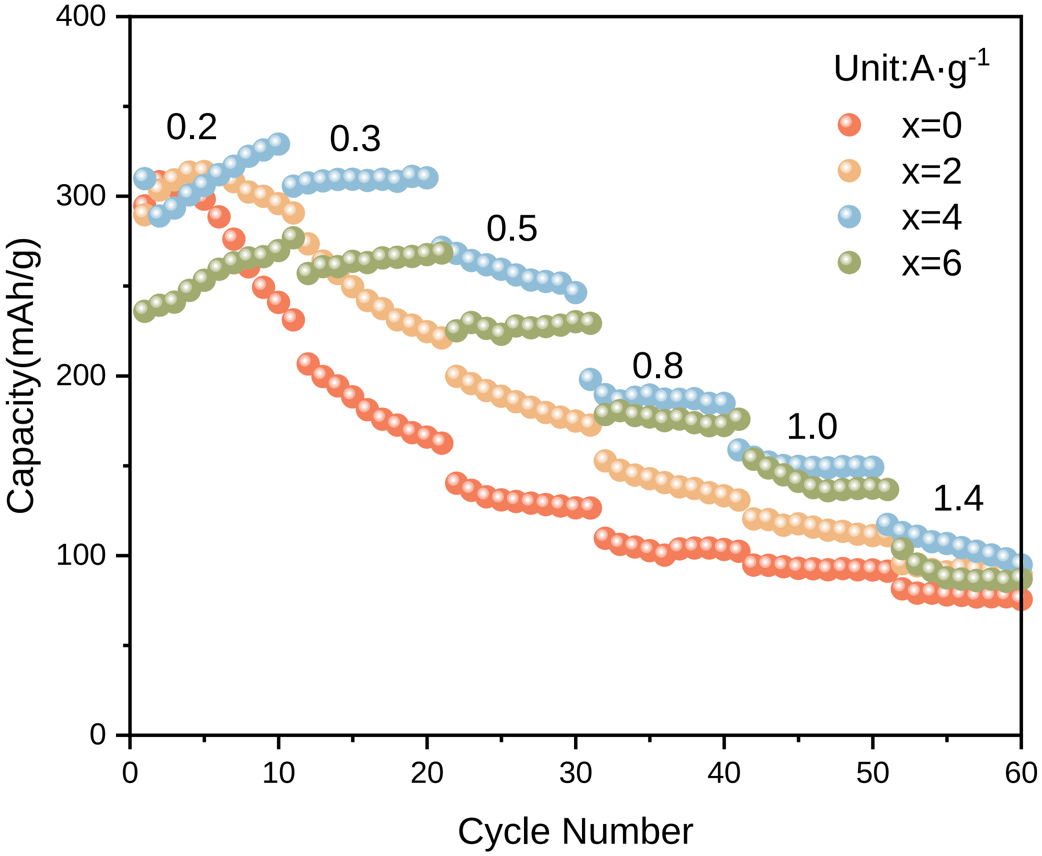

Figure S25 Rate performances at different current densities from 0.2 $A \cdot g^{-1}$ to 1.4 $A \cdot g^{-1}$.

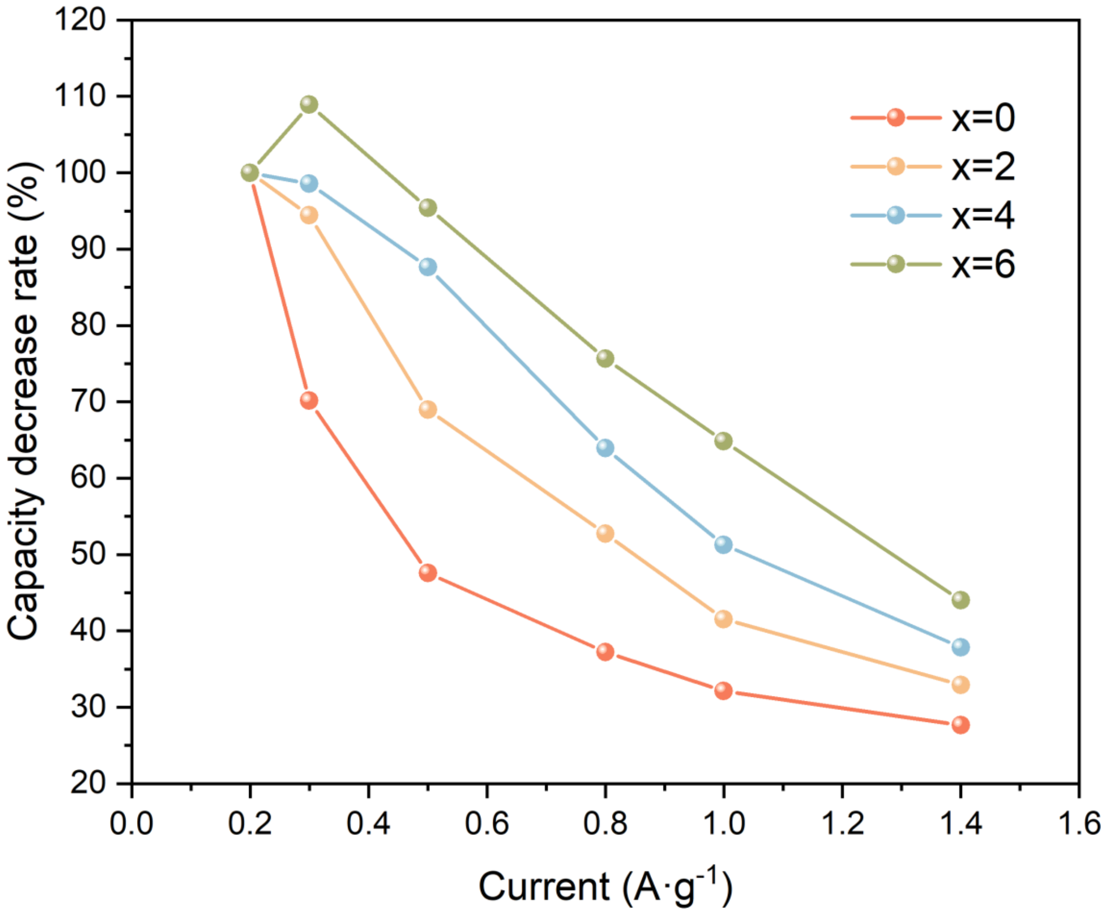

Figure S26 Capacity decrease rate with increased currents of different cells.

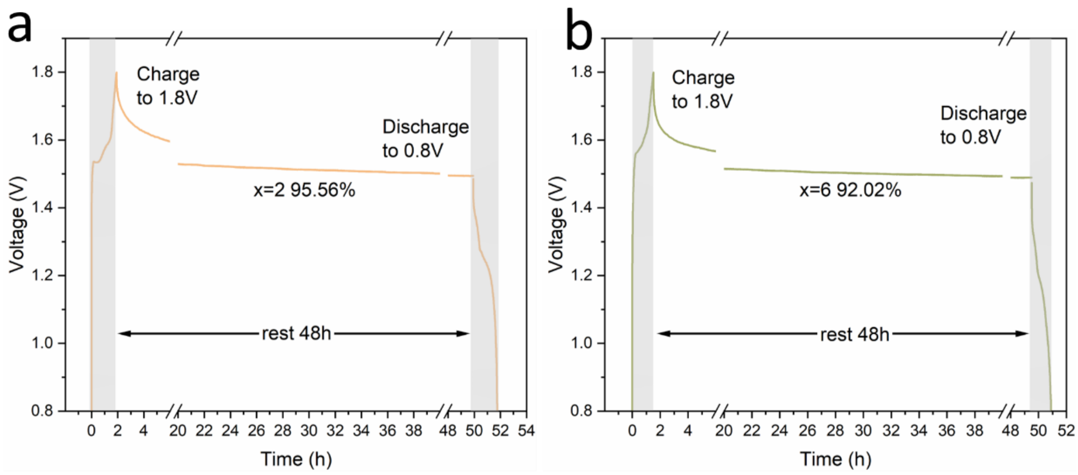

Figure S27 Self-discharge curves of different electrolytes. (a) x=2. (b) x=6.

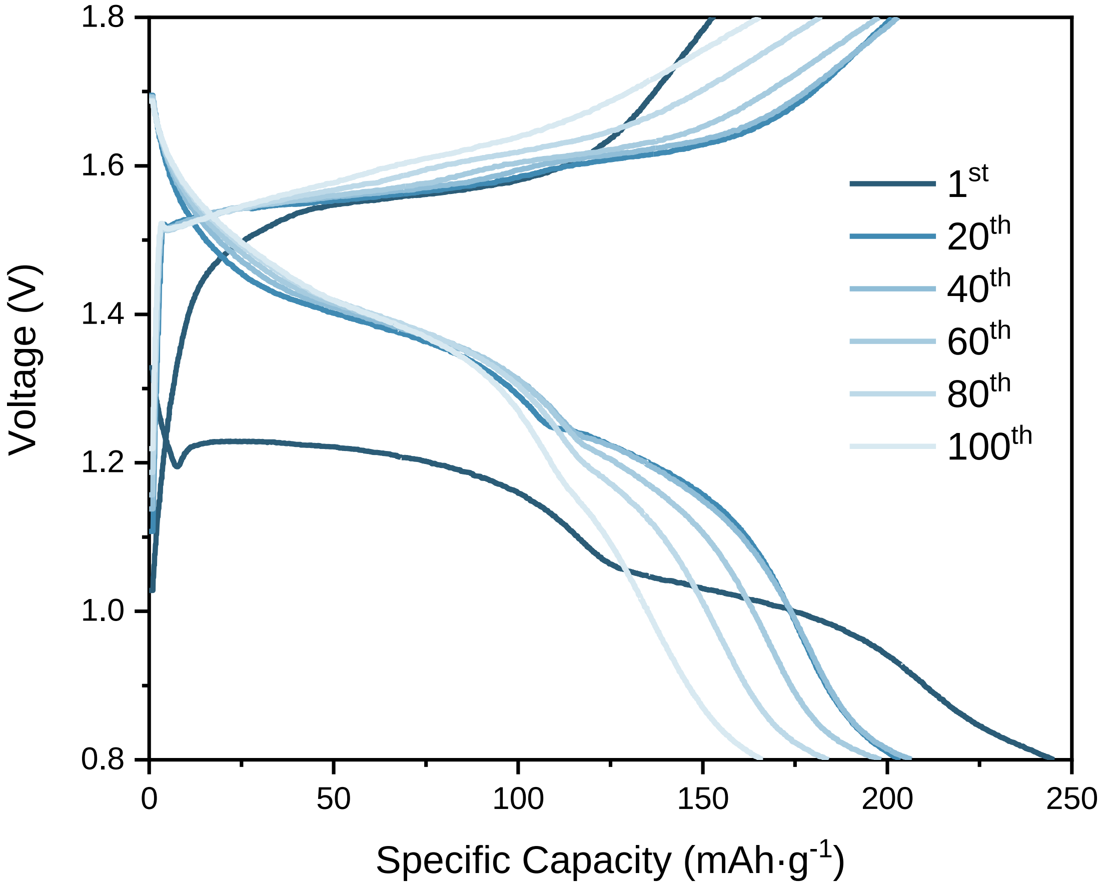

Figure S28 Galvanostatic charge and discharge profiles of soft-pack battery in x=4 electrolytes at 0.3 $A \cdot g^{-1}$.

Supplementary Note 1

In **Figure S27**, compared to the x=0 electrolyte, the initial discharge current of the oxidation peak of the x=4 electrolyte gradually increases as the scan rate rises. In general, during discharge, the cations migrate from the cathode to the anode. In the x=4 electrolyte, on the other hand, Zn^{2+} is bound by the urea at the cathode interface, making the migration of Zn^{2+} difficult and thus leading to a rise in the initial discharge current.

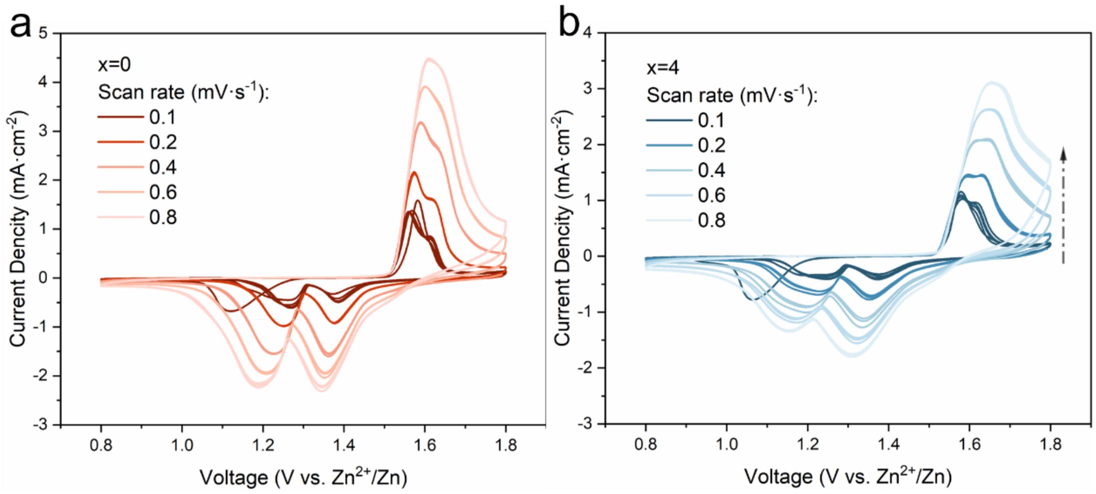

Figure S29 CV curves of different cells at different current densities from 0.1 mV·s^{-1} to 0.8 mV·s^{-1}.

Supplementary Note 2

As mentioned in the article, QEE has a binding effect on Zn^{2+}. Therefore, tests were carried out on Zn anode to demonstrate that QEE does not have a side effect on Zn anode. We tested the performance of Zn||Zn symmetric cells (Figure S30a). After 200 hours of cycling, the QEE-containing symmetric cell still remains stable and has a lower polarization voltage than the aqueous electrolyte (x=0). In contrast, the polarization in Figure 5b and c occurs only from the tenth turn of the battery cycle, which is far less than 200 hours. This is sufficient evidence that the polarization of the battery is not related to the anode, but is caused by the cathode. And we have chosen the voltage-capacity curve of the symmetrical battery at hours 100-102 (Figure S30b). When x=4, symmetrical cells have a much smaller voltage polarization, with a polarization voltage of only 0.048V (Figure S30c). This shows that QEE does not inhibit the kinetics of zinc anode, but on the contrary, it promotes the kinetics of Zn^{2+} deposition. In FigureS30d, the LSV curve proves that when x=4, the QEE does not increase the risk of hydrogen precipitation, but on the contrary, the hydrogen precipitation potential decreases.

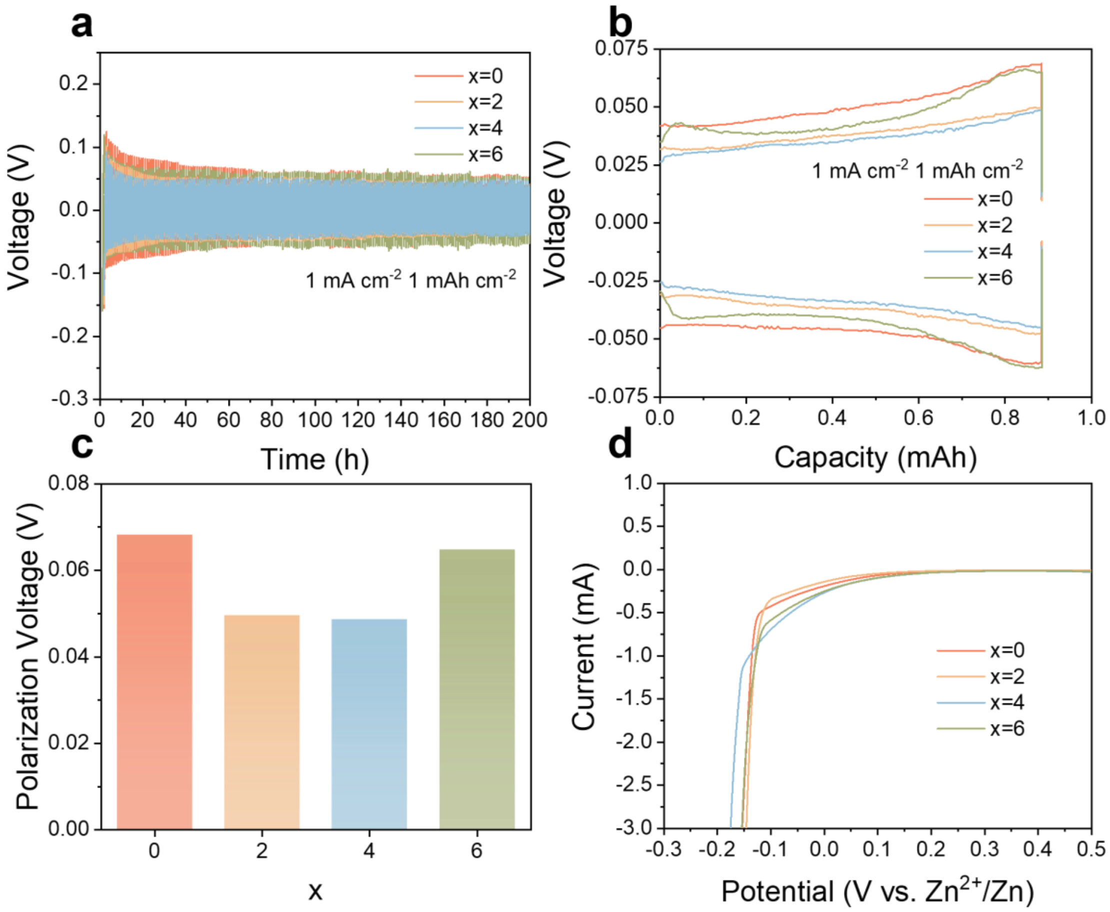

Figure S30 (a) galvanostatic cycling performances of Zn||Zn symmetrical cells. (b) Voltage-capacity curve for Zn||Zn symmetrical cells. (c) Polarization voltage for different electrolytes in (b). (d) LSV curves of different electrolytes.

Table S1 Binding energy between the various groups.

Binding Form	Binding Energy (eV)
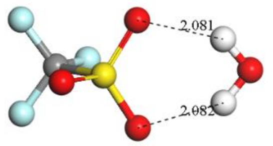	**-0.72**
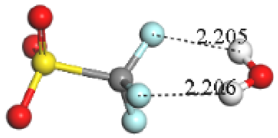	-0.41
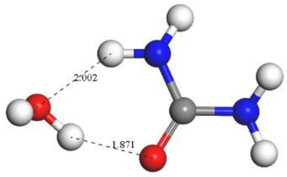	**-0.64**
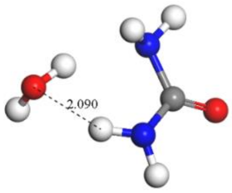	-0.49
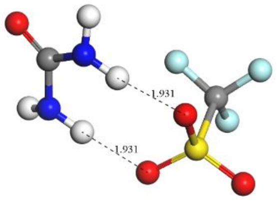	**-1.07**
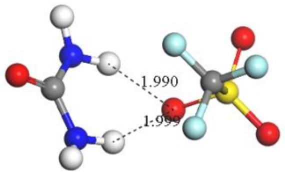	-1.01

Notice that the H-F structure is unstable and cannot be obtained as a stable structure. And the lower the energy, the more stable the structure.

Table S2 Energy barriers for the determining steps in the desolvation of Zn^{2+} and Mn^{2+} in different electrolytes

Electrolyte	Ion	Step	Energy barrier (eV)
x=0	Mn^{2+}	H_2O to OTf^-	-15.94
		OTf^- to H_2O	-10.72
	Zn^{2+}	H_2O to OTf^-	-15.94
		OTf^- to H_2O	-10.05
x=4	Mn^{2+}	H_2O to OTf^-	-15.94
		OTf^- to H_2O	-10.72
	Zn^{2+}	H_2O to OTf^- to urea	-12.35
		H_2O to urea to OTf^-	-15.94
		OTf^- to H_2O to urea	-9.02
		OTf^- to urea to H_2O	-9.02
		urea to OTf^- to H_2O	-10.28
		urea to H_2O to OTf^-	-15.94

Table S3. Number of groups of different components in different electrolyte in MD

	Zn^{2+}	OTf^-	Mn^{2+}	SO_4^{2}-	H_2O	$CO(NH_2)_2$
x=0	400	800	50	50	6837	0
x=2	400	800	50	50	5681	400
x=4	400	800	50	50	4704	800
x=6	400	800	50	50	3796	1200

METHODS

α-MnO_2 cathode synthesis

In a typical synthesis of procedure, 15 ml of $KMnO_4$ (0.1 M) aqueous solution was added to 15 ml of $MnSO_4$ (0.15 M) with vigorous stirring for 30 minutes. Place the mixture in 50 ml Teflon-lined autoclaves and heat at 160 □ for 12 h. The precipitate was collected by vacuum suction filter, washed with deionized water, and dried at 60 □ in a vacuum for 12 h.

Characterization

The crystallographic phase, bonding state, and chemical composition of the as-prepared products was studied by X-ray power diffraction (Rigaku D/max2500, Cu Kα radiation, λ = 1.54178 Å), Fourier transform infrared spectroscopy (FTIR, Nicolet6700) and Nuclear Magnetic Resonance (NMR, Bruker-400M). The composition of the electrolyte in NMR was expressed as the concentration of $Zn(OTf)_2$ + the concentration of urea. 2+4 means that the electrolyte contains 2 M $Zn(OTf)_2$ and 4 M urea, e.g. The nanoscale morphologies were characterized by scanning electron microscopy (SEM, MIRA3 TESCAN) and Transmission electron microscope (TEM, Titan G2 60-300). The valence states were characterized by X-ray photoelectron spectroscopy (XPS, ESCALAB 250Xi). The electrolyte structure was studied by Raman (HORIBA HR Evolution, 532 nm wavelength) and Fourier transform infrared spectroscopy (FTIR, Nicolet6700). The zeta-potential and cathode interface adsorption were studied by nanoparticle size and potential analyzer (Malvern PANalytical, Zetasizer Nano ZSE) and QCM-D (QSense Analyzer).

Electrochemical tests

The cathode electrodes were prepared by casting a slurry mixed with sample powder (70 wt.%), Ketjen black (20 wt.%), and polyvinylidene fluoride (PVDF, 10 wt.%) onto the stainless-steel wire mesh (SSWM, 400 orders) with the area of ~1.13 cm^2, and then dried at 80 °C for 12 h in a vacuum. The electrochemical performances were tested by assembling the CR2016 coin-type cells with the Zn foil anode (≥ 99.9 %), glass fiber separator, α-MnO_2 cathode and various electrolytes. If not otherwise stated, all electrolyte electrolytes in the article have compositions of 2 M $Zn(OTf)_2$ + x M Urea + 0.25 M $MnSO_4$. The value of x will be given in the article and in the Figure S as appropriate. Cyclic voltammetry (CV) and electrochemical impedance spectrum (EIS) measurements from 0.01 Hz to 100 kHz were conducted on an electrochemical workstation (CHI660E, CHI Instruments). Galvanostatic charge/discharge (GCD) and cycling performance were carried out on the LAND-2001A testing system. The voltage range for all electrochemical tests without special instructions is 0.8-1.8 V (vs. Zn^{2+}/Zn). The potential-temperature curves were measured in a three-electrode system consisting of the α-MnO_2 cathode (working electrode), Platinum sheet (counter electrode) and Ag/AgCl (reference electrode).

Computational details

The Gibbs free energies of solvated molecule were optimized by DFT calculation, using Gaussian 16 program package.[1] The B3LYP functional,[2] 6-31G (d, p) basis set and van der Waals force correction were adopted in these calculations.[3] The GROMACS software package was used for the molecular dynamics (MD) simulations.[4] The general Amber force field (GAFF) force field was employed to running for the electrolyte samples with different carboxylate anions.[5] The simulations were performed at 298.15 K. The volume of the box is 332.11 nm^3. The number of different groups in different electrolytes is shown at Supplementary Table 3.

REFERENCES

[1] M. J. Frisch, G. W. Trucks, H. B. Schlegel, G. E. Scuseria, M. A. Robb, J. R. Cheeseman, G. Scalmani, V. Barone, G. A. Petersson, H. Nakatsuji, X. Li, M. Caricato, A. V. Marenich, J. Bloino, B. G. Janesko, R. Gomperts, B. Mennucci, H. P. Hratchian, J. V. Ortiz, A. F. Izmaylov, J. L. Sonnenberg, Williams, F. Ding, F. Lipparini, F. Egidi, J. Goings, B. Peng, A. Petrone, T. Henderson, D. Ranasinghe, V. G. Zakrzewski, J. Gao, N. Rega, G. Zheng, W. Liang, M. Hada, M. Ehara, K. Toyota, R. Fukuda, J. Hasegawa, M. Ishida, T. Nakajima, Y. Honda, O. Kitao, H. Nakai, T. Vreven, K. Throssell, J. A. Montgomery Jr., J. E. Peralta, F. Ogliaro, M. J. Bearpark, J. J. Heyd, E. N. Brothers, K. N. Kudin, V. N. Staroverov, T. A. Keith, R. Kobayashi, J. Normand, K. Raghavachari, A. P. Rendell, J. C. Burant, S. S. Iyengar, J. Tomasi, M. Cossi, J. M. Millam, M. Klene, C. Adamo, R. Cammi, J. W. Ochterski, R. L. Martin, K. Morokuma, O. Farkas, J. B. Foresman, D. J. Fox, Wallingford, CT, **2016**.

[2] A. D. Becke, *The Journal of Chemical Physics* **1993**, *98*, 5648-5652.

[3] U. Essmann, L. E. Perera, M. L. Berkowitz, T. A. Darden, H.-C. Lee, L. G. J. J. o. C. P. Pedersen, **1995**, *103*, 8577-8593.

[4] M. J. Abraham, T. Murtola, R. Schulz, S. Páll, J. C. Smith, B. Hess, E. Lindahl, *SoftwareX* **2015**, *1-2*, 19-25.

[5] a) E. Pohjolainen, X. Chen, S. Malola, G. Groenhof, H. Häkkinen, *Journal of Chemical Theory and Computation* **2016**, *12*, 1342-1350; b) J. Wang, R. M. Wolf, J. W. Caldwell, P. A. Kollman, D. A. Case, *Journal of Computational Chemistry* **2004**, *25*, 1157-1174.
